# Identification of Tropical Plant Extracts That Extend Yeast Chronological Life Span

**DOI:** 10.3390/cells10102718

**Published:** 2021-10-11

**Authors:** Mandy Mun Yee Kwong, Jee Whu Lee, Mohammed Razip Samian, Habibah A. Wahab, Nobumoto Watanabe, Eugene Boon Beng Ong

**Affiliations:** 1 Institute for Research in Molecular Medicine (INFORMM), Universiti Sains Malaysia—USM, Penang 11800, Malaysia; libragirl1015@gmail.com (M.M.Y.K.); jeewhu@student.usm.my (J.W.L.); 2 USM-RIKEN International Centre for Ageing Science (URICAS), Universiti Sains Malaysia—USM, Penang 11800, Malaysia; razip.samian@gmail.com (M.R.S.); habibahw@usm.my (H.A.W.); nwatanab@riken.jp (N.W.); 3 School of Biological Sciences, Universiti Sains Malaysia—USM, Penang 11800, Malaysia; 4 School of Pharmaceutical Sciences, Universiti Sains Malaysia—USM, Penang 11800, Malaysia; 5 Bio-Active Compounds Discovery Research Unit, RIKEN Center for Sustainable Resource Science, Saitama 351-0198, Japan

**Keywords:** ageing, life span, plant extracts, stress response, *Saccharomyces cerevisiae*

## Abstract

Certain plant extracts (PEs) contain bioactive compounds that have antioxidant and lifespan-extending activities on organisms. These PEs play different roles in cellular processes, such as enhancing stress resistance and modulating longevity-defined signaling pathways that contribute to longevity. Here, we report the discovery of PEs that extended chronological life span (CLS) in budding yeast from a screen of 222 PEs. We identified two PEs, the leaf extracts of *Manihot esculenta* and *Wodyetia bifurcata* that extended CLS in a dose-dependent manner. The CLS-extending PEs also conferred oxidative stress tolerance, suggesting that these PEs might extend yeast CLS through the upregulation of stress response pathways.

## 1. Introduction

Ageing is a biological process that occurs with gradual structural and functional changes [1,2] in all living organisms that eventually leads to death [3]. With the passage of time, cellular and body systems start to deteriorate with the accumulation of cellular damages and toxic substances in cells [4]. Various studies on ageing have been performed with different model organisms such as *Mus musculus* (mouse), *Caenorhabditis elegans* (worm), *Drosophila melanogaster* (fruit fly), and *Saccharomyces cerevisiae* (budding yeast). Model organisms have a relatively shorter generation time and life spans that range from weeks to months, and their smaller sizes are advantageous for laboratory high throughput ageing experiments. Additionally, unicellular organisms such as yeast allow us to study the molecular mechanisms of ageing and senescence at the cellular level [5].

Cellular chronological ageing is a type of ageing that occurs in postmitotic, non-replicating cells. In yeast, chronological life span (CLS) is defined as the length of time that a non-dividing yeast cell is viable and can re-enter the cell cycle to reproduce new cells upon the introduction of a new growth medium [6,7,8]. CLS assay of non-dividing yeast cells is a model that resembles the ageing process in postmitotic cells such as neurons and muscle cells [9,10], thus the yeast model can provide useful insights into cellular ageing.

Natural phytochemicals found in various plant extracts (PEs) were shown to have pharmacological value towards living organisms and contribute to life span extension in model organisms without affecting the essential biological functions of cells [11,12,13]. It is widely thought that the antioxidant activity of the phytochemicals is one of the main contributors to anti-ageing [14]. For example, it was reported that the antioxidant activities of aqueous polyphenols such as tannic acid and glucosides in *Rosa rugosa* [15], and the carotenoids, chlorophylls and flavonoids in the fruit pulp of *Dipteryx alata* [16,17] contribute to the life span extension of *C. elegans*.

Several screenings of PEs were performed to study their effects on CLS and their roles in ageing-related cellular processes and mechanisms in budding yeast *S. cerevisiae*. For instance, a screen of 53 PEs discovered 15 extracts, respectively, from berries of *Serenoa repens*, aerial parts of *Hypericum perforatum*, leaves of *Ilex paraguariensis*, leaves of *Ocimum tenuiflorum*, whole plant of *Solidago virgaurea*, fruits of *Citrus sinensis*, whole plant of *Humulus lupulus*, grape skins of *Vitis vinifera*, whole plant of *Andrographis paniculata*, roots of *Hydrastis canadensis*, seeds of *Trigonella foenum-graecum*, root barks of *Berberis vulgaris* leaves, flowers and stems of *Crataegus monogyna*, leaves of *Taraxacum erythrospermum*, and the whole plant of *Ilex paraguariensis* that prolong yeast CLS. These 15 PEs were shown to play roles in cellular processes such as enhancing mitochondrial respiration, reducing cellular reactive oxygen species (ROS) level, protecting cellular genetics and proteins from oxidative damage and promoting oxidative and thermal stress tolerance for extending CLS [18]. In addition, a screen of 35 PEs identified six extracts, respectively, from root and rhizome of *Cimicifuga racemosa*, root of *Valeriana officinalis* L., whole plant of *Passiflora incarnate* L., leaf of *Ginkgo biloba*, seed of *Apium graveolens* L., and bark of *Salix alba* that increase yeast CLS via different involvement in the longevity-defined cellular processes as described formerly for the 15 PEs [19]. Moreover, these six CLS-extending PEs are also involved differently in highly conserved longevity-regulating signalling pathways such as target of rapamycin complex 1 (TORC1) pathway [20,21], protein kinase A (PKA) pathway [21], and Pkb-activating kinase homolog (PKH1/2) pathway [22], which prolong ageing, and sucrose non-fermenting (SNF1) pathway [23] and autophagy (ATG) pathway [24,25], which delay ageing [26].

In this study, we sought to identify tropical plants extracts that can extend yeast CLS. As a preliminary step towards the discovery of phytochemicals that may regulate ageing, we used a 96-well plate-based CLS outgrowth assay to screen 222 crude methanolic PEs from 175 species of 70 plant families. We initially identified seven candidate PEs that extended yeast CLS and further characterised the top three potential PEs in terms of their dose-dependent activity and stress tolerance during chronological ageing. We show that two of the PEs have dose-dependent effects and that the PEs potentially confer oxidative stress tolerance during chronological ageing. Our findings reveal the presence of ageing regulating molecules in non-herbal or medicinal plants and highlight the case for continuous bioprospecting from tropical plants.

## 2. Materials and Methods

### 2.1. Yeast Strains, Media and Growth Conditions

The *S.* *cerevisiae* strain 1783 (*MATa can1 his4 leu2-3,112 trp1-1 ura3-52*) (National BioResource Project, Hiroshima, Japan) was used in this study. Yeast cells were cultured at 30 °C with shaking at 200 rpm in Yeast Peptone Dextrose (YPD) medium (10 g/L yeast extract, 20 g/L peptone, and 20 g/L dextrose) (HIMEDIA, Mumbai, India) for general growth. Synthetic complete (SC) medium consisting of 2% (*w*/*v*) glucose (HIMEDIA, Mumbai, India), 0.67% (*w*/*v*) yeast nitrogen base with ammonium sulphate (HIMEDIA, Mumbai, India), complete supplement mixture without tryptophan (HIMEDIA, Mumbai, India) and supplemented tryptophan (HIMEDIA, Mumbai, India) was used for ageing cultures. The concentrations of amino acids in SC medium are listed in Supplementary Table S1.

### 2.2. Preparation of Plant Extract (PE) Library

A total of 222 crude methanolic PEs derived from 175 species from 70 families of plants was obtained from the USM-RIKEN Joint Laboratory for Bioprobe Discovery. A total of 20 milligrams of PEs were dissolved in 1 mL of 100% dimethyl sulfoxide (DMSO) to a final concentration of 20 mg/mL in 1.5 mL microcentrifuge tubes, respectively, and stored at −20 °C. The working stocks of PEs were prepared at 10 mg/mL by diluting 10 μL of 20 mg/mL PEs each with 10 μL of DMSO in 96-well microplates. The microplate plates were sealed with adhesive film and stored upright at −20 °C. All the PEs were labelled with a code that matches their locations on the 96-well plate. A PE library with information of each PE (local name, scientific name, family name, plant organ, and PE code) is listed (Supplementary Table S2).

### 2.3. Chronological Life Span Assay

The CLS assay for screening PEs was based on a 96-well plate CLS assay we had established previously [27]. First, the yeast strain 1783 was streaked on YPD agar and incubated for 2 days. A single colony was selected and grown overnight in a Bijou bottle with 1 mL of YPD medium at 30 °C with shaking at 200 rpm. A total of 3 colonies were selected as 3 biological replicates of PE-treated culture or control culture. An amount of 1.8 μL overnight culture was transferred to each well of 96-well flat-bottom microplate (IWAKI) containing 176.4 μL of SC medium and 1.8 μL of PEs at final concentration of 100 μg/mL. Culture of cells grown in SC medium with 1.8 μL of DMSO served as the control. The mixtures were suspended several times with multichannel pipette to ensure even distribution of PEs in the liquid culture. The cultures in the microplate were incubated at 30 °C without agitation throughout the 8 days of ageing. The day after an initial 48 h of growth was defined as day 0 of the stationary phase. On day 0, 2, 4, and 6 of the stationary phase, an aliquot of 2 μL of each replicate of ageing culture was transferred to a new 96-well flat bottom microplate (IWAKI) containing 98 μL YPD medium to screen the PEs. After transferring the aliquots of cultures to a new microplate, the aged cultures were re-incubated at 30 °C.

In the primary screen, the outgrowths of yeast cultures in the new 96-well microplate were monitored by measuring the absorbance at a wavelength of 600 nm (A_600_) every 6 h until 24 h using Bio Microplate Reader HiTS (Cosmo Bio, Tokyo, Japan). From the primary screen, the PEs that increased the relative absorbance of culture (average absorbance value of each PE-treated culture at the 12th h divided by average absorbance value of the control culture at 12th h), which was higher than that of the control culture throughout the ageing period (Supplementary Tables S3–S6, Figure S6), were selected for the secondary screen.

In the secondary and confirmatory screens, the outgrowths of yeast cultures in the new 96-well microplate were monitored by measuring the absorbance (A_600_) every 30 min until 24 h using Bio Microplate Reader HiTS (Cosmo Bio, Tokyo, Japan). The cultures in the 96-well microplate were incubated at 30 °C, with continuous shaking at 210 rpm in the reader [27]. The PEs that increased the survival percentage of cultures throughout the ageing period in the secondary screen (Supplementary Figure S9) were selected for the confirmatory screen. Survival percentage of each replicate of aged culture was determined by the formula: S_n_ = $\frac{1}{2^{\left(\frac{\Delta t_{n}}{\delta_{r}}\right)}}\times 100\%$, whereby S_n_ = survival percentage on day n, ∆t_n_ = time shift from day 0 to day n at A_600_ 0.3, determined from the linear regression equation of natural logarithm of A_600_ and δ_r_ = doubling time of each replicate. This δ_r_ was calculated from the maximal slope of the semilog plot of absorbance against time and was defined as the average of at least three δ values for that replicate. Doubling times (δ) were calculated between every consecutive pair of absorbance values between 0.2 and 0.5, by the formula: δ = $\frac{\ln\left(2\right)}{\left(\frac{\ln\left(A_{2}\right)-\ln\left(A_{1}\right)}{t_{2}\;-\;t_{1}}\right)}$, whereby A_2_ and A_1_ are successive absorbance values while t_1_ and t_2_ are the times of consecutive pairs of respective absorbance values. The survival percentage of each replicate of each PE-treated culture was then averaged. A graph of average survival percentage against ageing period (day) was plotted [6]. From the survival percentage graph, survival integral (SI) was calculated as the area under the curve (AUC) by applying mathematical trapezium rule: SI = $\sum_{2\;}^{n\;}\left(\frac{S_{n-2}+S_{n}}{2}\right)({\mathrm{day}}_{n}-{\mathrm{day}}_{n-2})$, whereby day_n_ is the day of outgrowth measurement (day Identification of tropical plant extracts that extend yeast chronological lifespan 2, 4, 6 in this study) [6].

### 2.4. Dose-Dependent Effects of Plant Extracts on Yeast Chronological Life Span

A single colony of yeast strain 1783 was selected from YPD agar and grown overnight in a Bijou bottle with 1 mL of YPD medium at 30 °C with shaking at 200 rpm. Three colonies were selected as 3 biological replicates of each culture. An amount of 500 μL overnight culture was centrifuged for 2 min at 10,000 rpm, washed twice and resuspended in 500 μL SC medium. An aliquot of 10 μL cell suspension was transferred to 988 μL SC medium containing 2 μL PEs (*M. esculenta*, *W. bifurcata*, *T. divaricata* leaf extracts) at different final concentrations of 0.1, 1, 5, 10, 50, 100, 200 μg/mL for each PE in a Bijou bottle [27]. As a control, 10 μL cell suspension was transferred to 988 μL SC medium containing 2 μL DMSO in a Bijou bottle. The cells were incubated and aged at 30 °C with continuous shaking at 200 rpm throughout the experiment. On day 0, 2, 4, and 6 of the stationary phase, an aliquot of 2 μL of each aged culture was transferred into a 96-well flat-bottom microplate (IWAKI) containing 98 μL YPD medium. The outgrowths of yeast cultures in the 96-well microplate were monitored by measuring the A_600_ every 30 min until 24 h using Bio Microplate Reader HiTS (Cosmo Bio, Tokyo, Japan). Survival percentages and survival integrals of the cultures were calculated using the formulae stated earlier in the chronological life span assay. The analysis of significant differences in survival integrals was performed using one-way ANOVA with the Tukey test. Differences at *p* < 0.05 were considered statistically significant.

### 2.5. Stress Assay

A single colony of yeast strain 1783 was selected from YPD agar and grown overnight in a Bijou bottle with 1 mL of YPD medium at 30 °C with shaking at 200 rpm. At least 3 biological replicates of each PE-treated culture or control culture were prepared from different single colonies. An amount of 500 μL overnight culture was centrifuged for 2 min at 10,000 rpm, washed twice and resuspended in 500 μL SC medium. An aliquot of 10 μL cell suspension was transferred to 988 μL SC medium containing 2 μL PE (*M. esculenta* or *W. bifurcata* leaf extract) at a final concentration of 50 and 10 μg/mL, respectively, in a Bijou bottle [27]. As a control, 10 μL cell suspension was transferred to 988 μL SC medium containing 2 μL DMSO in a Bijou bottle. The cells were incubated and aged at 30 °C with continuous shaking at 200 rpm throughout the experiment. On day 0, 4, 6 and 10 of the stationary phase, an aliquot of 50 μL of each aged culture was washed twice and resuspended in 50 μL sterile distilled water. The aged culture suspensions were exposed to oxidative stress by being treated with 3 mM hydrogen peroxide (H_2_O_2_) for 1 h in the dark at 30 °C with shaking at 200 rpm. After H_2_O_2_ treatment, the cells were washed twice and resuspended in sterile distilled water. For thermal stress exposure, an amount of 50 μL of each aged culture suspension was incubated in a water bath at 55 °C for 20 min and were cooled at 30 °C for 30 min. A 10-fold serial dilution (10^−2^, 10^−3^, 10^−4^, 10^−5^) was conducted by diluting the non- and stress-treated cultures in distilled water. An amount of 10 μL 10-fold serially diluted non- and stress-treated cultures were spotted on YPD agar in duplicate. The agar plates were incubated for 2 days at 30 °C. Colonies of the strains formed after 2 days were observed. For outgrowth measurement, an amount of 2 μL of non- or stress-treated culture was transferred to a 96-well flat-bottom microplate (IWAKI) containing 98 μL YPD medium on day 0, 4, 6 and 10. The outgrowths of yeast cultures in the 96-well microplate were monitored by measuring the A_600_ every 30 min until 24 h using Bio Microplate Reader HiTS (Cosmo Bio, Tokyo, Japan). Survival percentages of the cultures were calculated using the formulae stated earlier in the chronological life span assay.

## 3. Results

### 3.1. Seven PEs Extend Chronological Life Span in Yeast

To determine the effects of the PEs on yeast CLS, we used an adapted chronological life span assay for 96-well plate screening as previously reported [27]. Briefly, the yeast cells were treated with PEs at a final concentration of 100 μg/mL and were grown and aged in 96-well microplates containing SC media. To measure cell survival, an aliquot of ageing culture (on day 0, 2, 4 and 6) was transferred to a 96-well microplate containing YPD media for outgrowth. The absorbances of outgrowth cultures were recorded by a microplate reader for result analysis.

**Figure 1:**
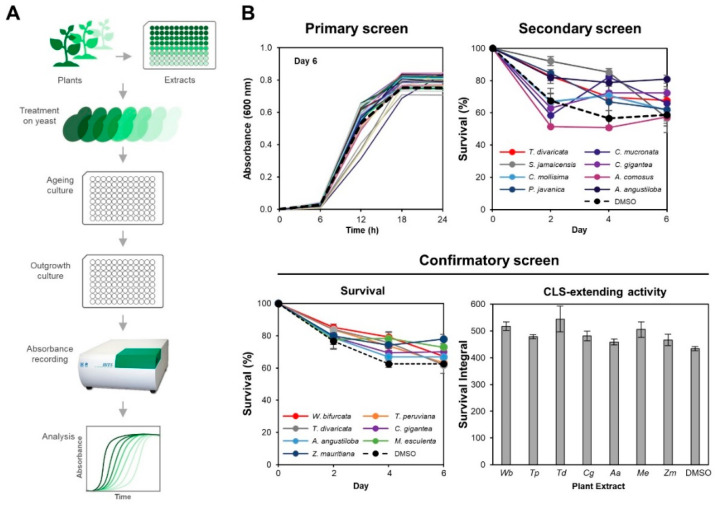
Screening for plant extracts (PEs) that extend yeast chronological life span (CLS). (**A**) Overview of the screening for PEs that extend *S. cerevisiae* CLS. Yeast cells of strain 1783 treated with various PEs or DMSO control were grown and aged in a 96-well microplate containing SC media. An aliquot of each aged culture was transferred to a new 96-well microplate containing fresh YPD medium to screen the PEs. The outgrowths of yeast cells were monitored by measuring their absorbances (A_600_) using Bio Microplate Reader HiTS on alternate days. Growth curves of absorbance against time of outgrowth were plotted. (**B**) Representative results of the primary, secondary and confirmatory screens. In the primary screen, the absorbance values of 222 PEs-treated cultures at the 12th h from the outgrowth curves (Supplementary Figures S1–S5, Tables S3–S6) were used to calculate the relative absorbance values. The relative absorbance values were calculated by dividing the PE-treated cultures’ average outgrowth absorbance readings at the 12th h to that of the DMSO control culture. Three biological replicates of each culture were prepared. In the secondary screen, the survival percentages of 45 PEs-treated cultures selected from the primary screen (Supplementary Figure S9) were derived from the outgrowth curves (Supplementary Figures S7 and S8). Error bars represent the standard errors of means (SEM) of three biological replicates. In the confirmatory screen, the survival percentages and survival integrals of seven PEs-treated cultures selected from the secondary screen were derived from the outgrowth curves (Supplementary Figure S10). Error bars represent the SEM of three biological replicates.

From a total of 222 PEs (Supplementary Table S2) screened in the primary screen, 45 PEs-treated cultures recorded higher relative absorbances than that of the control culture throughout the ageing period (Supplementary Figure S6) and were selected for secondary screening. Among the 45 PEs, seven PEs (*Wodyetia bifurcata* leaf extract, *Thevetia peruviana* leaf extract, *Tabernaemontana divaricata* leaf extract, *Calotropis gigantea* leaf extract, *Alstonia angustiloba* leaf extract, *Manihot esculenta* leaf extract, *Ziziphus mauritiana* leaf extract) were found to have an overall higher survival than the control culture during chronological ageing (Supplementary Figure S9 and Figure 1B). The CLS extensions by the seven PEs were further verified in a third confirmatory screen. The survivals of the seven PEs-treated cultures on individual days 2, 4 and 6 were 1.11, 1.27 and 1.07 (*W. bifurcata*); 1.09, 1.18 and 1.01 (*T. peruviana*); 1.09, 1.21 and 0.99 (*T. divaricata*); 1.03, 1.11 and 1.12 (*C. gigantea*); 1.03, 1.07 and 1.07 (*A. angustiloba*); 1.01, 1.25 and 1.16 (*M. esculenta*), and 1.04, 1.18 and 1.24 (*Z. mauritiana*), -times of the survival of control culture, respectively (Figure 1B). The overall survival integrals (SI), which was the area under curve (AUC) of the survival curves, of the cultures were 1.19 (*W. bifurcata*); 1.10 (*T. peruviana*); 1.26 (*T. divaricata*); 1.11 (*C. gigantea*); 1.06 (*A. angustiloba*); 1.16 (*M. esculenta*), and 1.08 (*Z. mauritiana*), -times of the survival integral of control culture, respectively (Figure 1B). The top three CLS-extending PEs (*T. divaricata*, *W. bifurcata*, and *M. esculenta*) were selected for further characterisation in the dose-dependent and stress assays.

### 3.2. M. esculenta and W. bifurcata Extracts Provide Dose-Dependent Effects in Extending Yeast CLS

To evaluate the dose-dependent effects of PEs on yeast CLS, the cells were treated with *M. esculenta*, *W. bifurcata* or *T. divaricata* leaf extract at different final concentrations of 0.1, 1, 5, 10, 50, 100, 200 μg/mL and aged in Bijou bottles containing SC media. On day 0, 2, 4 and 6, an aliquot of the PE-treated ageing culture was transferred to a 96-well microplate containing YPD media for outgrowth measured by a microplate reader.

**Figure 2:**
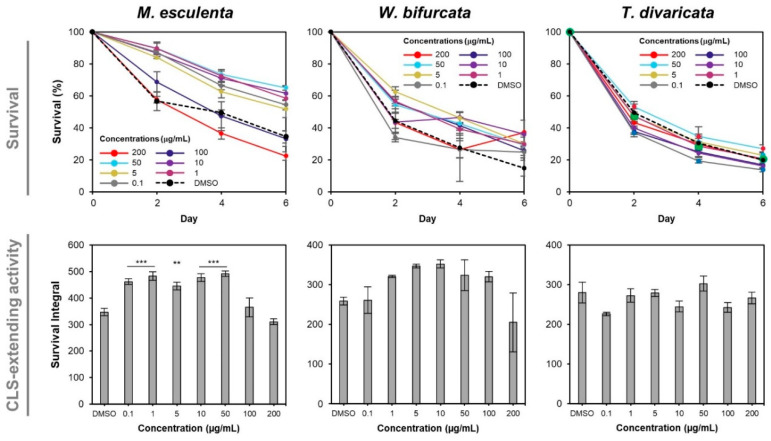
Dose-dependent effects of *M. esculenta*, *W. bifurcata* and *T. divaricata* extracts on yeast CLS. Yeast cells of strain 1783 treated with different concentrations of *M. esculenta*, *W. bifurcata* or *T. divaricata* leaf extract, or DMSO control were chronologically aged in Bijou bottles containing SC media until day 6. An aliquot of each aged culture was transferred to a 96-well microplate containing YPD medium for outgrowth on day 0, 2, 4 and 6. The survival percentages and survival integrals of the cultures were derived from the outgrowth curves (Supplementary Figure S11). Tukey test was performed (IBM SPSS Statistics software). The survival integrals of yeast cells treated with *M. esculenta* only at 0.1, 1, 5, 10, 50 μg/mL were significantly different from that of the DMSO control cells (** *p* < 0.01; *** *p* < 0.001). Error bars represent the SEM of three biological replicates.

The survival of yeast cells treated with *M. esculenta* leaf extract at ≤50 μg/mL was beyond 50%, higher than that of the control throughout the ageing period while the survival of yeast cells treated with *M. esculenta* leaf extract at ≥100 μg/mL was less than 50%, lower than the control on day 4 and 6 (Figure 2). To compare the extract’s CLS-extending activity across different concentrations tested, the survival integral, which was the AUC of the survival curve, was constructed. The survival integrals of yeast cells treated with *M. esculenta* leaf extract at ≤50 μg/mL were higher than that of the control culture (** *p* < 0.01; *** *p* < 0.001) while the survival integrals of yeast cells treated with *M. esculenta* extract at ≥100 μg/mL were not significantly different compared to that of the control cultures. The yeast cells treated with *M. esculenta* extract at 50 μg/mL had the highest survival integral, which was 1.42-times of that of the control culture (Figure 2).

The survivals of yeast cultures treated with *W. bifurcata* leaf extract ranged from 1 μg/mL to 100 μg/mL were higher than that of the DMSO control throughout the ageing period while the survivals of yeast cultures treated with *W. bifurcata* leaf extract at 0.1 μg/mL and 200 μg/mL were lower than that of the control on day 2 and 4. The comparison of CLS-extending activity between groups of cultures treated with *W. bifurcata* leaf extract at different concentrations and the control was at a weak significant difference, *p* = 0.07. Nevertheless, there is a trend of increasing survival integrals of the yeast cultures treated with *W. bifurcata* leaf extract plateauing in the range of 5 μg/mL to 10 μg/mL, followed by a decrease with increasing concentrations of the extract above 50 μg/mL when compared to the DMSO control. The 10 μg/mL of *W. bifurcata* extract was found to have the highest survival integral of 1.36-times the control (Figure 2).

Intriguingly, the survivals of yeast cultures treated with the *T. divaricata* extract were not significantly different at all the tested concentrations compared to the DMSO control. This may be due to the different culture conditions compared to the screening stage. Therefore, the *T. divaricata* extract was excluded for further characterisation. Overall, the *M. esculenta* and *W. bifurcata* leaf extracts at 50 μg/mL and 10 μg/mL respectively were found to result in the highest survival integral and were selected for further characterisation.

### 3.3. M. esculenta and W. bifurcata Extracts Contribute to Oxidative Stress Tolerance during Chronological Ageing

To evaluate the stress response of yeast cultures treated with *M. esculenta* or *W. bifurcata* leaf extracts, we used the optimal concentration of 50 μg/mL and 10 μg/mL, respectively, identified from the dose-dependent assay. The PE-treated cultures were aged in Bijou bottles containing SC media as before, then the aged cultures were subjected to oxidative stress by the addition of 3 mM hydrogen peroxide (H_2_O_2_) or to thermal stress by incubation at 55 °C. Spot assays were performed to observe the growth phenotype and an outgrowth assay was also used to quantify the cell survival upon post-stress treatment.

**Figure 3:**
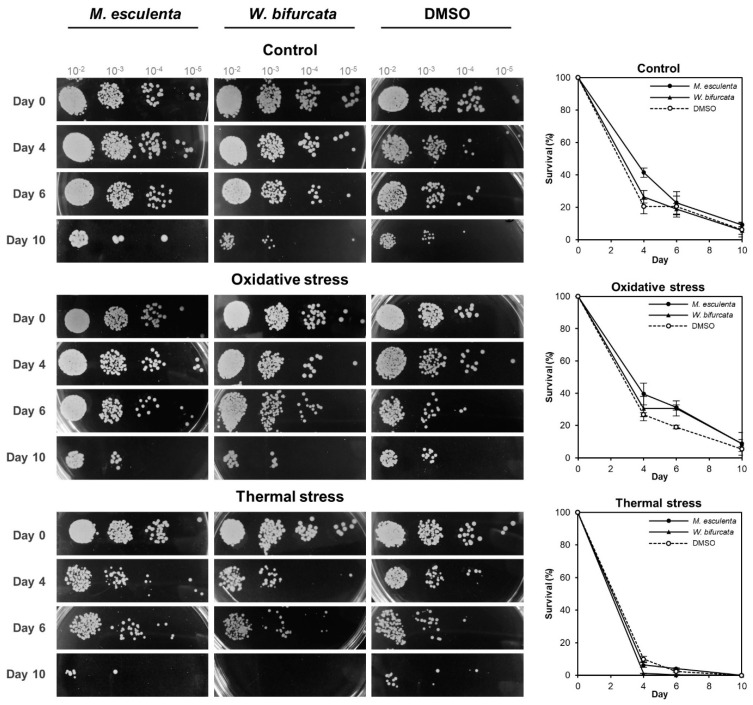
Oxidative and thermal stress responses of PEs-treated yeast and control yeast during chronological ageing. Yeast cultures of strain 1783 treated with *M. esculenta* or *W. bifurcata* leaf extract at optimal final concentrations of 50 μg/mL and 10 μg/mL, respectively, or DMSO control were chronologically aged in Bijou bottles until day 10. At each age point, a small volume of yeast cells was transferred out of the ageing culture and was exposed to oxidative stress by being treated with 3 mM H_2_O_2_ for 1 h at 30 °C or thermal stress at 55 °C for 20 min before being spotted in 10-fold serial dilutions (10^−2^, 10^−3^, 10^−4^, 10^−5^) on YPD agar. Quantitative outgrowth CLS analysis was carried out simultaneously to determine the survival of cultures aged with *M. esculenta* or *W. bifurcata* leaf extracts after oxidative and thermal stress exposure. An aliquot of each non- or stress-treated culture was transferred to a 96-well microplate containing YPD medium for outgrowth on day 0, 4, 6 and 10. The survival percentages of the cultures were derived from the outgrowth curves (Supplementary Figure S12). Error bars represent the SEM of at least three (three to six) biological replicates.

In the spot assay, which was a semi-quantitative assay (Figure 3), we observed that the yeast cultures treated with *M. esculenta* leaf extract had a lower survival compared to the non-treated respective yeast cultures by forming less colonies at 10^−4^ and 10^−5^ dilutions starting from day 0 and forming colonies only up to 10^−3^ dilution on day 10 after oxidative or thermal stress exposure.

In the case of the yeast cultures treated with *W. bifurcata* leaf extract, they had lower survival compared to the non-treated yeast cultures by forming fewer colonies at 10^−4^ and 10^−5^ dilutions starting from day 0 and forming colonies only up to 10^−4^ dilution on day 6 after oxidative stress and forming colonies only up to 10^−3^ dilution on day 4, up to 10^−4^ dilution on day 6, and no colonies were formed on day 10 after thermal stress exposure. For the DMSO control cultures, they had lower survival compared to the non-stress treated respective yeast cultures by forming less colonies at 10^−4^ and 10^−5^ dilutions on day 0, 6 and 10 and forming colonies only up to 10^−4^ dilution on day 0 after oxidative stress exposure and forming fewer colonies at 10^−3^ and 10^−4^ dilution starting from day 4 after thermal stress exposure. Overall, the growth phenotypes of the PEs-treated or DMSO control cultures were affected negatively after stress exposure during chronological ageing.

To quantitate the cell survival upon stress exposure, we also performed an outgrowth kinetics assay (Figure 3, right panels). We previously demonstrated that cell number was directly related to absorbance (OD_600_) [27]. In the non-stress treated, *M. esculenta* leaf extract extended CLS in budding yeast throughout the ageing period agreeing with dose-dependent result, however the *W. bifurcata* extract did not extend CLS beyond day 6. Both *M. esculenta* and *W. bifurcata* leaf extracts contributed to oxidative stress tolerance with a higher survival percentage than the DMSO control culture. However, they did not appear to contribute to thermal stress tolerance. These results indicate that *M. esculenta* and *W. bifurcata* leaf extracts may potentially be involved in the cellular oxidative stress response pathway to extend CLS in budding yeast.

## 4. Discussion

Various plants are known to contain ageing-delaying phytochemicals. Here, we report the identification of two PEs that have not been reported before that can extend *S. cerevisiae* life span. Specifically, we first developed a semi-high-throughput 96-well plate-based CLS assay, screened 222 PEs from 70 plant families, and identified two PEs that can extend yeast CLS possibly by conferring protection against oxidative stress.

*S. cerevisiae* has been widely used as a model organism for the discovery of PEs and small molecules that can extend CLS by using various cell viability protocols. For example, *Melannurca Campana* apple extracts were identified to extend yeast CLS through the microcolony scoring method [28]. The yeast CLS extension by pigmented Hom Dang rice bran extracts was determined using a methylene blue-stained cell counting method [29]. While those methods are useful in their respective studies, the laborious and time-consuming methods of microscopic colony count do not allow for parallel screening of multiple PEs simultaneously. The microplate-based yeast ageing and screening assay using the outgrowth method is a practical option that allows the identification of candidate PEs for further verification.

Our CLS screen first identified seven candidate PEs (*W. bifurcata* leaf extract, *T. peruviana* leaf extract, *T. divaricata* leaf extract, *C. gigantea* leaf extract, *A. angustiloba* leaf extract, *M. esculenta* leaf extract, and *Z. mauritiana* leaf extract) that could extend yeast CLS. These seven PEs with the presence of phytochemicals such as flavonoids, tannins, caratenoids, terpenoids, saponins and alkaloids, have been reported to exert various beneficial biological activities such as antibacterial activity, antiproliferative effect against cancer cell lines, antioxidant activity and so on in vitro and in vivo (Table 1).

Among the seven candidate PEs, the three leaf extracts of *M. esculenta*, *W. bifurcata*, and *T. divaricata* extended yeast CLS the most. Next, we further evaluated the dose-dependent effects of the top three CLS-extending PEs during chronological ageing. Among these three PEs, *M. esculenta* and *W. bifurcata* leaf extracts were dose-dependent in their extension of yeast CLS. However, *T. divaricata* leaf extracts did not show the same effect. This could be due to the different ageing culture incubation methods (non-shaking microplate-based or shaking bottle-based methods) in both assays.

Ageing can be caused by environmental stress [45]. Oxidative stress, which is a major contributor to ageing, occurs when ROS accumulate in cells. ROS such as hydrogen peroxide (H_2_O_2_), superoxide anion (O_2_^−^) and hydroxyl free radical (OH·) can cause oxidative damage of biomacromolecules [46,47]. We found that *M. esculenta* and *W. bifurcata* leaf extracts conferred oxidative stress tolerance to the ageing cells. *M. esculenta* extracts had been reported to possess antioxidant activity against lipid peroxidation and mild capability in capturing free radicals [48]. Additionally, *M. esculenta* extract contains carotenoid and flavonoid [42], which are well-known antioxidants in protecting cells from harmful ROS [49,50,51,52]. *W. bifurcata* extract also contains flavonoid [30], which acts as an antioxidant as well [51,52]. Flavonoid compounds consist of hydroxyl groups, which the hydrogens and electrons can be donated to ROS superoxide anion then further reduced into unharmed water and oxygen molecules [53]. Therefore, both *M. esculenta* and *W. bifurcata* extracts likely extended yeast CLS through the presence of antioxidant bioactive compounds that protected yeast cells from oxidative stress during chronological ageing. This agrees with studies that showed that PEs from apple, citrus peel and pigmented rice bran delay ageing by contributing resistance against oxidative stress in *S. cerevisiae* [28,29,54].

Thermal stress can cause cell death as the cells experience downregulated thermal stress response and thus accumulate damaged proteins and misfolded protein during ageing [45]. It was suggested that PEs could possibly extend yeast CLS through various longevity-relating cellular activities such as elevating oxidative and thermal stress tolerance [19]. However, both *M. esculenta* and *W. bifurcata* extracts did not confer thermal tolerance under the conditions that we tested (55 °C for 20 min). Furthermore, the survivals of the thermal-stressed cells were reduced by more than 50% compared to the non-stressed control.

In summary, we identified two PEs, *M. esculenta* and *W. bifurcata*, that extended yeast CLS possibly through the conferment of oxidative stress tolerance from carotenoid and flavonoid compounds that are present in the extracts. Further studies are needed to determine the effects of these PEs on various aspects of cellular processes such as the regulation of longevity-defined pathways (TOR and PKA pathways), mitochondrial respiration and lipid peroxidation in order to understand and elucidate the specific effects these PEs may have on longevity.

## Figures and Tables

**Table 1:** Phytochemical profiles and previously reported biological activities of seven PEs were identified from the CLS assay.

PE (Organ)	Phytochemical	Biological Activity	Reference
*Wodyetia bifurcate* (leaf)	Methanol and butanol extracts contain triterpene, flavonoids, benzenoid and polyphenol	Weak cytotoxicity against human liver Hep-G_2_ cancer cell line	[30]
*Thevetia peruviana* (leaf)	Contains alkaloids, cardiac glycosides, flavonoids, polyphenols, saponins and tannins	Antibacterial activity against foodborne microorganisms; treatment for a diarrhoea-induced rat model	[31,32]
*Tabernaemontana divaricate* (leaf)	Diverse in alkaloids and non-alkaloids	Mild insecticides against crop pests; folk medicine; mimics the effect of acetylcholinesterase inhibitors towards Alzheimer’s disease	[33,34,35]
*Calotropis gigantean* (leaf)	Hydro-alcoholic extracts contain flavonoid, tannins, alkaloids, and steroids	Traditional medicine; antifungal against *Candida* sp.; antibacterial activity towards *Escherichia coli*., *Pseudomonas aeruginosa* and *Bacillus cereus*; antiproliferative effect against human cancer cell lines (MCF7, MDA-MB-231, and HeLa); antioxidant activity	[36,37,38,39]
*Alstonia angustiloba* (leaf)	Ethanol extracts contain alkaloids	Weak antiproliferative against human cancer cell lines (MCF7, MDA-MB-231, and HeLa)	[39,40]
*Manihot esculenta* (leaf)	Ethanol extracts contain terpenoids, flavonoids, carotenoids and tannins	In vivo anti-inflammatory on rat model with paw oedema; contains antipyretic activity, which mimics the effect of paracetamol; anthelmintic activity against gastrointestinal parasite *Haemonchus contortus*	[41,42]
*Ziziphus mauritiana* (stem)	Various solvent extraction contains alkaloids, flavonoids, tannins, carotenoids, saponins, steroids, triterpenoids and anthraquinones	Traditional medicine; in vivo anti-ulcers against aspirin and ethanol ulcers in mice model; detoxification of silica-induced toxicity in the liver of Albino rats; antibacterial against pathogens such as *Salmonella typhi*, *Bacillus subtilis* and *Staphylococcus aureus*	[12,43,44]

## Data Availability

The data presented in this study are available on request from the corresponding author.

## Supplementary Materials

The following are available online at https://www.mdpi.com/article/10.3390/cells10102718/s1, Table S1: final concentrations of amino acids in SC medium, Table S2: list of 222 plant extracts used for primary CLS assay screen, Table S3: absorbance values (A_600_) of yeast cultures treated with PEs (P1 A1 until P1 H11) at the 12th h from day 0 to day 6 in primary screen, Table S4: absorbance values (A_600_) of yeast cultures treated with PEs (P2 A1 until P2 D11) at 12th h from day 0 to day 6 in primary screen, Table S5: absorbance values (A_600_) of yeast cultures treated with PEs (P2 E1 until P2 H12) at the 12th h from day 0 to day 6 in primary screen, Table S6: absorbance values (A_600_) of yeast cultures treated with PEs (P3 A1 until P3 C12) at the 12th h from day 0 to day 6 in primary screen, Figure S1: CLS outgrowth curves of yeast treated with PEs (P1 A1 until P1 D12) in primary screen, Figure S2: CLS outgrowth curves of yeast treated with PEs (P1 E1 till P1 H11) in primary screen, Figure S3: CLS outgrowth curves of yeast treated with PEs (P2 A1 until P2 D11) in primary screen, Figure S4: CLS outgrowth curves of yeast treated with PEs (P2 E1 until P2 H12) in primary screen, Figure S5: CLS outgrowth curves of yeast treated with PEs (P3 A1 until P3 C12) in primary screen, Figure S6: relative absorbance values (A_600_) of 45 PEs-treated cultures, which are higher than that of the DMSO control cultures at the 12th h from day 0 to day 6 in primary screen, Figure S7: CLS outgrowth curves of yeast treated with PEs in secondary screen, Figure S8: CLS outgrowth curves of yeast treated with PEs in secondary screen, Figure S9: survival of yeast treated with 45 PEs in secondary screen, Figure S10: CLS outgrowth curves of yeast treated with seven PEs in confirmatory screen, Figure S11: CLS outgrowth curves of yeast treated with three PEs in dose-dependent assay, Figure S12: CLS outgrowth curves of yeast treated with two PEs in stress assays.
