# Supplementary material for: Identification of Tropical Plant Extracts That Extend Yeast Chronological Life Span
Source: Cells. 2021 Oct 11;10(10):2718. doi: 10.3390/cells10102718 (PMC8534465; doi:10.3390/cells10102718)
Supplement: Supplementary file 1 [file cells-10-02718-s001.zip › cells-1377734-supplementary.pdf]

Table S1. Final concentrations of amino acids in SC medium.

Amino acid	Final concentration in SC medium (mg/L)
Adenine	10
L-Arginine HCl	50
L-Aspartic acid	80
L-Histidine HCl	20
L-Isoleucine	50
L-Leucine	100
L-Lysine HCl	50
L-Methionine	20
L-Phenylalanine	50
L-Threonine	100
L-Tyrosine	50
Uracil	20
L-Valine	140
L-Tryptophan	140

Table S2. List of 222 plant extracts used for primary CLS assay screen.

No.	Local Name	Scientific Name	Family	Organ	PE Code
1	Rumput israel	*Asystasia gangetica*	Acanthaceae	Stem	P1 D11
2	Rumput israel	*Asystasia gangetica*	Acanthaceae	Leaf	P2 D11
3	Bisa ular	*Barleria lupulina*	Acanthaceae	Leaf	P2 A2
4	Belalai gajah	*Clinacanthus nutans*	Acanthaceae	Leaf	P1 G6
5	Ganda rusa	*Gendarussa vulgaris*	Acanthaceae	Leaf	P1 C11
6	Pecah beling	*Strobilanthes crispus*	Acanthaceae	Stem	P1 H9
7	Pecah beling	*Strobilanthes crispus*	Acanthaceae	Leaf	P2 B9
8	Pecah beling	*Strobilanthes crispus*	Acanthaceae	Stem	P2 C9
9	Balong ayam	*Celosia argentea*	Amaranthaceae	Other	P1 B7
10	Balong ayam	*Celosia argentea*	Amaranthaceae	Leaf	P1 H11
11	Spider Lily	*Lycoris radiata*	Amaryllidaceae	Whole	P2 A12
12	Gajus/Cashew	*Anacardium occidentale*	Anacardiaceae	Leaf	P1 F12
13	Mango	*Mangifera indica*	Anacardiaceae	Leaf	P2 G7
14	Durian belanda	*Annona muricata*	Annonaceae	Leaf	P1 D8
15	Asoka	*Polyalthia longifolia var. pendula*	Annonaceae	Leaf	P1 F1
16	Pegaga	*Centella asiatica*	Apiaceae	Leaf	P3 B6
17	Selom	*Oenanthe javanica*	Apiaceae	Leaf & Stem	P1 E7
18	Pokok pulai	*Alstonia angustiloba*	Apocynaceae	Leaf	P2 E10
19	Lembiaga	*Calotropis gigantea*	Apocynaceae	Leaf	P2 E6
20	Kemboja	*Plumeria acuminata*	Apocynaceae	Leaf	P1 H8
21	Susun kelapa/Crepe jasmine	*Tabernaemontana divaricate*	Apocynaceae	Leaf	P1 B12
22	Susun kelapa	*Tabernaemontana divaricate*	Apocynaceae	Stem	P2 B12
23	Yellow oleander	*Thevetia peruviana*	Apocynaceae	Leaf	P1 E11
24	Ekor naga/Dragon Tail	*Epipremnum pinnatum*	Araceae	Stem	P1 E9
25	Yellow aralia / Miagos bush	*Osmoxylon lineare*	Araliaceae	Leaf	P2 H12
26	Pokok Krismas/Star pine	*Araucaria heterophylla*	Araucariaceae	Leaf	P2 C10
27	Pokok dudar/Fishtail palm	*Caryota mitis*	Arecaceae	Leaf	P2 A10
28	Kelapa	*Cocos nucifera*	Arecaceae	Leaf	P2 F5
29	Pinang raja/Pinang merah	*Cyrrtostachys renda BL.*	Arecaceae	Leaf	P2 F9
30	Kelapa sawit/Oil palm	*Elaeis guineensis*	Arecaceae	Leaf	P2 G5
31	Palas kipas/Fan palm	*Licuala grandis*	Arecaceae	Other	P1 G9
32	Palas kipas / Fan palm	*Licuala grandis*	Arecaceae	Leaf	P3 B5
33	Palas	*Licuala grandis*	Arecaceae	Other	P3 B4
34	Palma ekor musang	*Wodyetia bifurcata*	Arecaceae	Leaf	P1 E10
35	Kelumpang telur/Carribean agave	*Agave angustifolia*	Asparagaceae	Leaf	P2 A6
36	Pokok Ti	*Cordyline fruticosa 'kiwi'*	Asparagaceae	Other	P1 A9
37	Kapal terbang	*Chromolaena odorata*	Asteraceae	Stem	P2 D5
38	Kapal terbang	*Chromolaena odorata*	Asteraceae	Other	P2 C5
39	Kapal terbang	*Eupatorium odoratum Linn*	Asteraceae	Leaf	P1 D12
40	Beluntas/Indian camphorweed	*Pluchea indica*	Asteraceae	Leaf	P2 G1
41	Nodeweed	*Synedrella nodiflora*	Asteraceae	Leaf	P1 D3
42	Tambak bukit/Iron Weed	*Vercenia cinerial*	Asteraceae	Leaf	P1 A12
43	Pucuk Paku	*Athyrium esculentum*	Athyriaceae	Leaf	P1 H7
44	Pucuk paku	*Athyrium esculentum*	Athyriaceae	Leaf	P2 G10
45	Daun Belimbing	*Averrhoa carambola*	Averrhoa	Leaf	P1 A6

46	Sakura Malaysia	*Tecoma tabebuia*	Bignoniaceae	Leaf	P1 B6
47	Nenas	*Ananas comosus*	Bromeliaceae	Other	P2 E8
48	Rhu	*Casuarina equisetifolia*	Casuarinaceae	Leaf	P2 C11
49	Manggis	*Garcinia mangostana*	Clusiaceae	Leaf	P1 E4
50	Asam gelugor	*Garcinia atroviridis*	Clusiaceae	Other	P2 B1
51	Asam gelugor	*Garcinia atroviridis*	Clusiaceae	Fruit	P2 C1
52	Black olive tree	*Bucida buceras*	Combretaceae	Leaf	P1 A4
53	Morning glory	*Turbina corymbosa*	Convolvulaceae	Leaf	P2 C8
54	Red button ginger	*Costus woodsonii*	Costaceae	Leaf	P2 B11
55	Setawar	*Kalanchoe pinnata*	Crassulaceae	Leaf	P2 G11
56	Timun	*Cucumis sativus*	Cucurbitaceae	Other	P2 F12
57	Rumput kyllingia putih	*Cyperus kyllingia*	Cyperaceae	Leaf	P1 E2
58	Rabbit's Foot Fern	*Davallia denticulata*	Davalliaceae	Leaf	P1 H3
59	Pokok mempelas	*Tetracera indica*	Dilleniaceae	Leaf	P2 D10
60	Belimbing tanah	*Tacca cristata*	Dioscoreaceae	Leaf	P1 F5
61	Seralat/Buah mentega	*Diospyros blancoi*	Ebenaceae	Leaf	P2 F11
62	Puding	*Codiaeum variegatum*	Euphobiaceae	Leaf	P1 B8
63	Ara tanah	*Euphorbia hirta*	Euphobiaceae	Leaf	P1 H1
64	Payung Indonesia/Sandbox tree	*Hura crepitans*	Euphobiaceae	Leaf	P2 A9
65	Mahang	*Macaranga tanarius*	Euphobiaceae	Leaf	P2 F7
66	Balik angin	*Mallothus biaceae*	Euphobiaceae	Leaf	P2 D1
67	Ubi Kayu/Cassava	*Manihot esculenta*	Euphobiaceae	Leaf	P2 G12
68	Akasia kuning	*Acacia auriculiformis*	Fabaceae	Leaf	P1 C2
69	Akasia kuning	*Acacia auriculiformis*	Fabaceae	Other	P2 A1
70	Saga/Red lucky seed	*Adenanthera pavonina*	Fabaceae	Leaf	P2 E11
71	Jering	*Archidendron jiringa (Jack) Nielsen*	Fabaceae	Other	P2 D4
72	Gelengang	*Cassia alata*	Fabaceae	Leaf	P3 A4
73	Gelengang	*Cassia alata*	Fabaceae	Stem	P3 A5
74	Gelengang	*Cassia alata*	Fabaceae	Flower	P3 A6
75	Gelengang	*Cassia alata*	Fabaceae	Other	P2 C3
76	Rajah kayu	*Cassia fistula*	Fabaceae	Leaf	P3 B9
77	Rajah kayu	*Cassia fistula*	Fabaceae	Stem	P3 B10
78	Rajah kayu	*Cassia fistula*	Fabaceae	Fruit	P3 B11
79	Cassia	*Cassia garettiana*	Fabaceae	Stem	P3 C7
80	Cassia	*Cassia garettiana*	Fabaceae	Fruit	P3 C8
81	Cassia	*Cassia garettiana*	Fabaceae	Leaf	P3 C9
82	Pink shower tree	*Cassia grandis*	Fabaceae	Leaf	P3 B7
83	Pink shower tree	*Cassia grandis*	Fabaceae	Stem	P3 B8
84	Gelengang	*Cassia obtusifolia L.*	Fabaceae	Flower	P3 A7
85	Glossy shower	*Cassia surattensis*	Fabaceae	Leaf	P3 C3
86	Glossy shower	*Cassia surattensis*	Fabaceae	Stem	P3 C4
87	Glossy shower	*Cassia surattensis*	Fabaceae	Fruit	P3 C5
88	Glossy shower	*Cassia surattensis*	Fabaceae	Roots	P3 C6
89	Orkid	*Clitoria fairchildiana*	Fabaceae	Other	P1 A8
90	Orkid	*Clitoria fairchildiana*	Fabaceae	Leaf	P3 B2
91	Giring-giring	*Crotalaria mucronata*	Fabaceae	Leaf	P2 E3
92	Nam-Nam	*Cynometra cauliflora*	Fabaceae	Leaf	P1 F7
93	Nam-nam	*Cynometra cauliflora*	Fabaceae	Stem	P2 D8

94	Tulang daing	*Millettia artopurpurea*	Fabaceae	Leaf	P1 H5
95	Semalu	*Mimosa pudica*	Fabaceae	Leaf & Stem	P1 H4
96	Petai kerayong	*Parkia javanica*	Fabaceae	Leaf	P2 E9
97	Petai	*Parkia speciosa*	Fabaceae	Leaf	P1 C3
98	Petai	*Parkia speciosa*	Fabaceae	Fruit	P2 D9
99	Jemerlang	*Peltophorum pterocarpum*	Fabaceae	Leaf	P2 C4
100	Jemerlang	*Peltophorum pterocarpum*	Fabaceae	Other	P1 G3
101	Mempari	*Pongamia pinnata*	Fabaceae	Other	P1 H6
102	Kacang botol	*Psophocarpus tetragonolobus*	Fabaceae	Leaf	P2 G4
103	Angsana	*Pterocarpus indicus*	Fabaceae	Leaf	P3 C11
104	Hujan-hujan/Pukul lima	*Samanea saman*	Fabaceae	Stem	P1 A11
105	Hujan-hujan/Pukul lima	*Samanea saman*	Fabaceae	Leaf	P2 F3
106	Christmas cassia	*Senna pendula*	Fabaceae	Leaf	P1 E8
107	Christmas cassia	*Senna pendula*	Fabaceae	Stem	P2 G2
108	Desert cassia	*Senna polyphylla*	Fabaceae	Leaf	P1 G5
109	Pokok Kembang Seberang	*Senna surattensis*	Fabaceae	Leaf	P1 E1
110	Geti/turi/hummingbird tree	*Sesbania grandiflora*	Fabaceae	Leaf	P2 D3
111	Asam jawa/Tamarind	*Tamarindus Indica*	Fabaceae	Other	P1 A5
112	Kacang parang/Broad bean	*Vicia faba*	Fabaceae	Leaf	P2 H4
113	Melinjau	*Gnetum gnemon*	Gnetaceae	Leaf	P1 B4
114	Pokok bangun-bangun	*Coleus amboinicus*	Lamiaceae	Leaf	P2 G9
115	Pudina	*Mentha x piperita*	Lamiaceae	Leaf	P2 H9
116	Selasih	*Ocimum basilicum*	Lamiaceae	Leaf & Stem	P1 C10
117	Tulasi/Holy Sacred Basil	*Ocimum tenuiflorum*	Lamiaceae	Leaf	P1 D4
118	Bebuas	*Premna foetida*	Lamiaceae	Leaf & Stem	P1 G1
119	Jati/Teak	*Tectona grandis*	Lamiaceae	Leaf	P2 B4
120	Halban	*Vitex Pubescens*	Lamiaceae	Leaf	P1 B3
121	Lemuni hitam	*Vitex trifolia var Negundo*	Lamiaceae	Leaf	P2 F6
122	Lemuni hitam	*Vitex trifolia var Negundo*	Lamiaceae	Stem	P2 G6
123	Teja/Medang tejar	*Cinnamomum iners*	Lauraceae	Leaf	P1 D2
124	Dedalu api	*Loranthus ferrugineus*	Loranthaceae	Leaf	P2 H2
125	Delima	*Punica granatum*	Lythraceae	Leaf	P1 A10
126	Berembang	*Sonneratia caseolaris*	Lythraceae	Leaf	P1 A7
127	Berembang	*Sonneratia caseolaris*	Lythraceae	Flower	P1 B5
128	Cempaka putih	*Michelia alba*	Magnoliaceae	Leaf	P1 D7
129	Durian	*Durio zibethinus*	Malvaceae	Leaf	P1 D5
130	Durian	*Durio zibethinus*	Malvaceae	Other	P2 A3
131	Bunga raya	*Hibiscus rosa-sinensis*	Malvaceae	Leaf	P2 C2
132	Bunga raya	*Hibiscus rosa-sinensis*	Malvaceae	Stem	P3 A2
133	Pokok kasah/Buddha coconut	*Pterygota alata*	Malvaceae	Leaf	P2 B10
134	Koko	*Theobroma cacao*	Malvaceae	Leaf	P2 B6
135	Bebaru/Portia tree	*Thespesia populnea (L.) Sol. ex correa*	Malvaceae	Leaf	P2 E1
136	Neem/Mambu	*Azadirachta indica*	Meliaceae	Leaf	P1 G11
137	Duku/langsat	*Lansium domesticum*	Meliaceae	Leaf	P1 F10
138	Kecapi/Sentul	*Sandoricum koetjape*	Meliaceae	Leaf	P1 C4
139	Kecapi/sentul	*Sandoricum koetjape*	Meliaceae	Stem	P2 E5
140	Sukun	*Artocarpus altilis*	Moraceae	Leaf	P1 B1
141	Cempedak	*Artocarpus integer*	Moraceae	Leaf	P1 F2
142	Mengkudu	*Ficus deltoidea*	Moraceae	Leaf	P3 B1

143	Bodhi/Ara Suci	*Ficus religiosa*	Moraceae	Leaf	P1 F11
144	Ara	*Ficus spp.*	Moraceae	Leaf	P3 A1
145	Remunggai/Kelor	*Moringa oleifera*	Moringaceae	Leaf	P1 D10
146	Remunggai/Kelor	*Moringa oleifera*	Moringaceae	Stem	P1 G4
147	Kerukup	*Muntingia calabura*	Muntingiaceae	Leaf	P1 E12
148	Ceri kampung/Kerukup siam	*Muntingia calabura*	Muntingiaceae	Leaf	P2 E2
149	Pala/Nutmeg	*Myristica fragrans*	Myristicaceae	Leaf	P1 C1
150	Pala	*Myristica fragrans*	Myristicaceae	Stem	P2 F8
151	Jambu Batu Kecil	*Psidium guajava Linn*	Myrtaceae	Leaf	P1 D1
152	Jambu laut	*Syzygium grandis*	Myrtaceae	Leaf	P3 A9
153	Jambu mawar	*Syzygium jambos*	Myrtaceae	Stem	P2 G3
154	jambu mawar	*Syzygium jambos*	Myrtaceae	Leaf	P2 H3
155	Paku larat/Giant Sword Fern	*Nephrolepis biserrata*	Nephrolepidaceae	Leaf	P3 B3
156	Bunga kertas/Bougainvillea	*Bougainvillea spp.*	Nyctaginaceae	Leaf	P1 H10
157	Mengkudu Siam	*Pisonia grandis*	Nyctaginaceae	Leaf	P2 B8
158	Melur	*Jasminum sambac*	Oleaceae	Leaf	P2 H7
159	Orkid merpati	*Dendrobium crumenatum*	Orchidaceae	Leaf	P1 A3
160	Belimbing Buluh/Bilimbi/Tree Sorrel	*Avverhoa bilimbi*	Oxalidaceae	Leaf	P1 F4
161	Belimbing buluh	*Averrhoa bilimbi*	Oxalidaceae	Other	P2 F1
162	Mengkuang	*Pandanus atrocarpus*	Pandanaceae	Leaf	P1 F6
163	Pandan kuning/Small screwpine	*Pandanus pygmaeus*	Pandanaceae	Leaf	P2 G8
164	Pandan Wangi	*Pandanus amaryllifolius*	Pandanaceae	Leaf	P2 H8
165	Letup-letup/running pop	*Passiflora foetida*	Passifloraceae	Leaf	P1 B9
166	Yellow turnera	*Turnera ulmifolia*	Passifloraceae	Stem	P3 C12
167	Bunga pukul lapan/Yellow alder	*Turnera ulmifolia*	Passifloraceae	Leaf	P2 B2
168	Cermai	*Phyllanthus acidus*	Phyllanthaceae	Leaf	P1 C6
169	Cermai	*Phyllanthus acidus*	Phyllanthaceae	Stem	P2 F2
170	Dukung anak	*Phyllanthus urinaria*	Phyllanthaceae	Leaf	P1 B2
171	Pine	*Pinus caribaea*	Pinaceae	Leaf	P1 E3
172	Pine	*Pinus caribaea*	Pinaceae	Other	P3 A3
173	Sireh cina/Pepper elder	*Peperomia pellucida*	Piperaceae	Leaf, Stem & Roots	P2 H11
174	Sireh	*Piper betle*	Piperaceae	Leaf	P1 E5
175	Kaduk	*Piper sarmentosum*	Piperaceae	Leaf	P1 C7
176	Kaduk	*Piper sarmentosum*	Piperaceae	Other	P2 A5
177	Serai	*Cymbopogon citratus*	Poaceae	Leaf	P1 C9
178	Rumput sambau/Goosegrass	*Eleusine indica*	Poaceae	Leaf	P1 G8
179	Buluh madu	*Gigantochloa albociliata*	Poaceae	Leaf	P1 F9
180	Tebu	*Saccharum officinale*	Poaceae	Leaf	P1 G10
181	Tebu hitam	*Saccharum officinarum*	Poaceae	Leaf	P2 C12
182	Ekor tupai	*Dacrydium beccarii*	Podocarpaceae	Leaf	P2 B3
183	Anggur laut/Sea grape	*Coccoloba uvifera*	Polygonaceae	Other	P1 F3
184	Tanduk rusa	*Phymatosorus scolopendria*	Polypodiaceae	Stem	P1 A1
185	Peria Pantai	*Colubrina asiatica*	Rhamnaceae	Leaf	P1 D9
186	Peria Pantai	*Colubrina asiatica*	Rhamnaceae	Stem	P1 B10
187	Bidara	*Ziziphus mauritiana*	Rhamnaceae	Leaf	P1 F8
188	Bidara	*Ziziphus mauritiana*	Rhamnaceae	Stem	P2 H1
189	Kopi	*Cafea canephora*	Rubiaceae	Leaf	P2 C6

190	Jenjarum (kuning)	*Ixora javanica 'yellow'*	Rubiaceae	Leaf	P1 C8
191	Jenjarum	*Ixora Siamensis*	Rubiaceae	Stem	P1 C5
192	Mas cotek/Mistlelog fig	*Morinda citrifolia*	Rubiaceae	Leaf	P3 A12
193	Mengkudu kecil	*Morinda elliptica*	Rubiaceae	Leaf	P2 A8
194	Janda kaya/Virgin tree	*Mussaenda philippica 'aurorae'*	Rubiaceae	Leaf	P2 A4
195	Limau bali/Pamelo	*Citrus grandis*	Rutaceae	Leaf	P1 B11
196	Limau kasturi/Calamondin	*Citrus microcarpa*	Rutaceae	Leaf & Stem	P1 G2
197	Limau bali	*Citrus grandis*	Rutaceae	Fruit	P2 C7
198	Limau purut	*Citrus hystrix*	Rutaceae	Fruit	P2 D7
199	Limau kasturi/Calamondin	Citrus microcarpa	Rutaceae	Fruit	P3 A10
200	Lemon	*Citrus x lemon*	Rutaceae	Leaf	P3 A11
201	Rokam manis	*Flacourtia jangomas*	Salicaceae	Leaf	P1 H2
202	Mata Kucing/Longan	*Dimocarpus longana*	Sapindaceae	Leaf	P1 E6
203	Rambutan	*Nephelium lappaceum*	Sapindaceae	Other	P2 F10
204	Rambutan	*Nephelium lappaceum*	Sapindaceae	Leaf	P1 C12
205	Bunga Tanjung/Spanish Cherry	*Mimusops elengi*	Sapotaceae	Stem	P1 A2
206	Tongkat ali	*Eurycoma longifolia*	Simaroubaceae	Stem	P3 B12
207	Terung Pipit	*Solanum torvum*	Solanaceae	Leaf	P1 G7
208	Terung pipit	*Solanum torvum*	Solanaceae	Fruit	P2 E12
209	Mahkota Dewa	*Phaleria macrocarpa*	Thymelaeaceae	Leaf	P1 D6
210	Cenerai	*Grewia tomentosa*	Tiliceae	Other	P2 D2
211	Jolok cacing	*Stachytarpheta jamaicensis*	Verbenaceae	Stem	P2 E4
212	Jolok cacing	*Stachytarpheta jamaicensis*	Verbenaceae	Leaf	P2 F4
213	Pokok bunga tahi ayam	*Lantana camara*	Verbenaceae	Other	P2 H10
214	Lokam gajah/gagnep	*Cayratia mollisima*	Vitaceae	Leaf	P2 E7
215	Temu lawak	*Curcuma zanthorrhiza*	Zingiberaceae	Leaf	P1 G12
216	Halia	*Zingiber officinale*	Zingiberaceae	Rhizome	P3 A8
217	Langkinang	*Zingiber spectabile*	Zingiberaceae	Leaf	P2 D6
218	Lengkuas (besar)/Greater galanga	*Alpinia galanga*	Zingiberaceae	Leaf	P2 H6
219	Lengkuas (besar)/Greater galanga	*Alpinia galanga*	Zingiberaceae	Stem	P2 A7
220	Kantan	*Etlingera elatior*	Zingiberaceae	Leaf	P2 B5
221	Lengkuas putih	*Alpinia conchigera*	Zingiberaceae	Leaf	P2 B7
222	Pokok pepanjat	*Liana spp.*	Unknown	Other	P3 C10

Table S3. Absorbance values (A_{600}) of yeast cultures treated with PEs (P1 A1 until P1 H11) at 12th h from day 0 to day 6 in primary screen.

Day	PE											
	**P1 A1**	**P1 A2**	**P1 A3**	**P1 A4**	**P1 A5**	**P1 A6**	**P1 A7**	**P1 A8**	**P1 A9**	**P1 A10**	**P1 A11**	**P1 A12**
0	0.515	0.491	0.486	0.516	0.465	0.471	0.510	0.483	0.518	0.558	0.509	0.563
2	0.629	0.672	0.643	0.615	0.618	0.645	0.662	0.633	0.602	0.556	0.533	0.499
4	0.508	0.588	0.511	0.515	0.547	0.511	0.566	0.523	0.496	0.477	0.444	0.417
6	0.433	0.534	0.433	0.431	0.396	0.474	0.460	0.418	0.505	0.446	0.430	0.450
	**P1 B1**	**P1 B2**	**P1 B3**	**P1 B4**	**P1 B5**	**P1 B6**	**P1 B7**	**P1 B8**	**P1 B9**	**P1 B10**	**P1 B11**	**P1 B12**
0	0.368	0.510	0.422	0.479	0.464	0.518	0.447	0.493	0.589	0.524	0.511	0.561
2	0.438	0.645	0.427	0.549	0.540	0.630	0.559	0.540	0.704	0.631	0.630	0.595
4	0.355	0.517	0.368	0.389	0.414	0.468	0.479	0.430	0.569	0.531	0.470	0.461
6	0.365	0.437	0.334	0.322	0.298	0.362	0.376	0.334	0.534	0.452	0.411	0.395
	**P1 C1**	**P1 C2**	**P1 C3**	**P1 C4**	**P1 C5**	**P1 C6**	**P1 C7**	**P1 C8**	**P1 C9**	**P1 C10**	**P1 C11**	**P1 C12**
0	0.427	0.372	0.351	0.317	0.329	0.334	0.322	0.376	0.461	0.441	0.316	0.448
2	0.570	0.528	0.407	0.415	0.456	0.336	0.415	0.475	0.499	0.530	0.437	0.554
4	0.354	0.423	0.251	0.320	0.353	0.236	0.217	0.371	0.264	0.269	0.251	0.377
6	0.268	0.312	0.136	0.291	0.395	0.278	0.235	0.359	0.194	0.277	0.296	0.347
	**P1 D1**	**P1 D2**	**P1 D3**	**P1 D4**	**P1 D5**	**P1 D6**	**P1 D7**	**P1 D8**	**P1 D9**	**P1 D10**	**P1 D11**	**P1 D12**
0	0.441	0.362	0.384	0.319	0.279	0.457	0.327	0.295	0.513	0.403	0.463	0.444
2	0.551	0.526	0.546	0.430	0.388	0.491	0.393	0.507	0.475	0.506	0.525	0.524
4	0.428	0.287	0.263	0.184	0.185	0.234	0.166	0.287	0.242	0.191	0.186	0.332
6	0.413	0.203	0.195	0.105	0.116	0.148	0.117	0.149	0.268	0.190	0.148	0.278
	**P1 E1**	**P1 E2**	**P1 E3**	**P1 E4**	**P1 E5**	**P1 E6**	**P1 E7**	**P1 E8**	**P1 E9**	**P1 E10**	**P1 E11**	**P1 E12**
0	0.228	0.568	0.578	0.550	0.534	0.639	0.568	0.575	0.685	0.690	0.635	0.700
2	0.664	0.688	0.708	0.573	0.624	0.709	0.680	0.638	0.730	0.735	0.704	0.731
4	0.640	0.667	0.712	0.580	0.650	0.684	0.672	0.632	0.681	0.697	0.673	0.686
6	0.571	0.604	0.632	0.497	0.581	0.602	0.612	0.526	0.519	0.589	0.590	0.540
	**P1 F1**	**P1 F2**	**P1 F3**	**P1 F4**	**P1 F5**	**P1 F6**	**P1 F7**	**P1 F8**	**P1 F9**	**P1 F10**	**P1 F11**	**P1 F12**
0	0.520	0.597	0.620	0.653	0.658	0.647	0.654	0.646	0.682	0.683	0.655	0.632
2	0.421	0.626	0.713	0.710	0.713	0.694	0.685	0.691	0.665	0.687	0.717	0.706
4	0.507	0.692	0.698	0.696	0.695	0.719	0.720	0.695	0.693	0.660	0.666	0.645
6	0.456	0.601	0.592	0.568	0.563	0.616	0.610	0.572	0.587	0.538	0.591	0.592
	**P1 G1**	**P1 G2**	**P1 G3**	**P1 G4**	**P1 G5**	**P1 G6**	**P1 G7**	**P1 G8**	**P1 G9**	**P1 G10**	**P1 G11**	**P1 G12**
0	0.471	0.493	0.549	0.516	0.489	0.506	0.438	0.530	0.589	0.580	0.553	0.532
2	0.457	0.622	0.693	0.723	0.662	0.621	0.578	0.704	0.588	0.721	0.658	0.649
4	0.393	0.420	0.598	0.621	0.531	0.585	0.459	0.581	0.426	0.549	0.563	0.589
6	0.323	0.414	0.516	0.545	0.498	0.526	0.425	0.506	0.414	0.530	0.454	0.459
	**P1 H1**	**P1 H2**	**P1 H3**	**P1 H4**	**P1 H5**	**P1 H6**	**P1 H7**	**P1 H8**	**P1 H9**	**P1 H10**	**P1 H11**	**DMSO**
0	0.550	0.577	0.581	0.609	0.631	0.560	0.614	0.585	0.607	0.590	0.605	0.569
2	0.564	0.626	0.665	0.683	0.732	0.618	0.666	0.640	0.710	0.663	0.633	0.696
4	0.571	0.548	0.546	0.557	0.596	0.475	0.556	0.520	0.573	0.533	0.608	0.577
6	0.470	0.440	0.432	0.447	0.538	0.447	0.454	0.497	0.476	0.438	0.466	0.535

Note: Green highlighted columns indicate absorbance values (A_{600}) which are higher than that of the DMSO control culture which is highlighted in blue. The PEs that have higher A_{600} throughout the ageing period (day 0, 2, 4, 6) are highlighted in orange.

Table S4. Absorbance values (A_{600}) of yeast cultures treated with PEs (P2 A1 until P2 D11) at 12th h from day 0 to day 6 in primary screen.

Day	PE											
	**P2 A1**	**P2 A2**	**P2 A3**	**P2 A4**	**P2 A5**	**P2 A6**	**P2 A7**	**P2 A8**	**P2 A9**	**P2 A10**		**P2 A12**
0	0.612	0.634	0.602	0.445	0.490	-0.002	0.630	0.623	0.658	0.622		0.640
2	0.597	0.598	0.578	0.595	0.597	-0.003	0.599	0.614	0.593	0.622		0.579
4	0.674	0.625	0.615	0.602	0.605	0.000	0.602	0.616	0.677	0.670		0.705
6	0.684	0.644	0.632	0.615	0.624	-0.001	0.613	0.642	0.663	0.679		0.716
	**P2 B1**	**P2 B2**	**P2 B3**	**P2 B4**	**P2 B5**	**P2 B6**	**P2 B7**	**P2 B8**	**P2 B9**	**P2 B10**	**P2 B11**	**P2 B12**
0	0.625	0.627	0.629	0.532	0.545	0.597	0.606	0.616	0.611	0.626	0.624	0.632
2	0.539	0.555	0.590	0.551	0.575	0.559	0.547	0.603	0.543	0.577	0.566	0.585
4	0.608	0.631	0.640	0.604	0.388	0.591	0.596	0.645	0.616	0.663	0.684	0.671
6	0.674	0.646	0.652	0.610	0.577	0.670	0.605	0.666	0.604	0.649	0.639	0.678
	**P2 C1**	**P2 C2**	**P2 C3**	**P2 C4**	**P2 C5**	**P2 C6**	**P2 C7**	**P2 C8**	**P2 C9**	**P2 C10**	**P2 C11**	**P2 C12**
0	0.598	0.521	0.426	0.573	0.435	0.562	0.649	0.557	0.528	0.594	0.531	0.602
2	0.490	0.467	0.228	0.501	0.425	0.440	0.508	0.483	0.476	0.474	0.394	0.532
4	0.566	0.496	0.275	0.518	0.385	0.455	0.561	0.531	0.517	0.554	0.450	0.547
6	0.545	0.524	0.312	0.552	0.481	0.525	0.567	0.585	0.499	0.513	0.438	0.659
	**P2 D1**	**P2 D2**	**P2 D3**	**P2 D4**	**P2 D5**	**P2 D6**	**P2 D7**	**P2 D8**	**P2 D9**	**P2 D10**	**P2 D11**	**DMSO**
0	0.581	0.590	0.569	0.548	0.331	0.563	0.540	0.582	0.857	0.547	0.539	0.584
2	0.410	0.479	0.510	0.528	0.299	0.494	0.479	0.522	0.506	0.442	0.443	0.571
4	0.408	0.549	0.573	0.518	0.248	0.549	0.507	0.556	0.617	0.580	0.558	0.635
6	0.387	0.567	0.605	0.585	0.348	0.575	0.534	0.576	0.616	0.574	0.526	0.654

Note: Green highlighted columns indicate absorbance values (A_{600}) which are higher than that of the DMSO control culture which is highlighted in blue. The PEs that have higher A_{600} throughout the ageing period (day 0, 2, 4, 6) are highlighted in orange.

Table S5. Absorbance values (A_{600}) of yeast cultures treated with PEs (P2 E1 until P2 H12) at 12th h from day 0 to day 6 in primary screen.

Day	PE											
	**P2 E1**	**P2 E2**	**P2 E3**	**P2 E4**	**P2 E5**	**P2 E6**	**P2 E7**	**P2 E8**	**P2 E9**	**P2 E10**	**P2 E11**	**P2 E12**
0	0.564	0.581	0.670	0.678	0.636	0.670	0.655	0.665	0.684	0.650	0.649	0.664
2	0.363	0.346	0.573	0.537	0.377	0.588	0.568	0.587	0.564	0.595	0.491	0.565
4	0.456	0.401	0.562	0.562	0.568	0.607	0.603	0.592	0.538	0.604	0.556	0.573
6	0.486	0.364	0.600	0.576	0.610	0.572	0.590	0.609	0.586	0.681	0.642	0.702
	**P2 F1**	**P2 F2**	**P2 F3**	**P2 F4**	**P2 F5**	**P2 F6**	**P2 F7**	**P2 F8**	**P2 F9**	**P2 F10**	**P2 F11**	**P2 F12**
0	0.675	0.693	0.691	0.686	0.659	0.643	0.552	0.708	0.710	0.001	0.597	0.670
2	0.522	0.585	0.606	0.427	0.356	0.358	0.346	0.584	0.585	0.001	0.555	0.583
4	0.573	0.605	0.609	0.575	0.569	0.468	0.475	0.609	0.597	-0.002	0.639	0.641
6	0.596	0.629	0.620	0.584	0.588	0.478	0.455	0.567	0.595	0.001	0.597	0.653
	**P2 G1**	**P2 G2**	**P2 G3**	**P2 G4**	**P2 G5**	**P2 G6**		**P2 G8**	**P2 G9**	**P2 G10**	**P2 G11**	**P2 G12**
0	0.578	0.566	0.564	0.391	0.314	0.513		0.503	0.588	0.559	0.616	0.574
2	0.543	0.539	0.544	0.520	0.532	0.498		0.525	0.610	0.585	0.551	0.549
4	0.425	0.412	0.447	0.420	0.446	0.417		0.411	0.488	0.494	0.464	0.535
6	0.494	0.411	0.421	0.473	0.473	0.447		0.482	0.419	0.391	0.431	0.597
	**P2 H1**	**P2 H2**	**P2 H3**	**P2 H4**	**P2 H6**	**P2 H7**	**P2 H8**	**P2 H9**	**P2 H10**	**P2 H11**	**P2 H12**	**DMSO**
0	0.583	0.569	0.487	0.555	0.516	0.493	0.476	0.577	0.575	0.537	0.569	0.439
2	0.599	0.601	0.493	0.543	0.528	0.533	0.610	0.562	0.545	0.562	0.696	0.505
4	0.454	0.475	0.390	0.437	0.419	0.448	0.465	0.485	0.390	0.484	0.577	0.421
6	0.549	0.461	0.373	0.472	0.409	0.468	0.515	0.413	0.333	0.476	0.535	0.493

Note: Green highlighted column(s) indicate absorbance values (A_{600}) which are higher than that of the DMSO control culture which is highlighted in blue. The PEs that have higher A_{600} throughout the ageing period (day 0, 2, 4, 6) are highlighted in orange.

Table S6. Absorbance values (A_{600}) of yeast cultures treated with PEs (P3 A1 until P3 C12) at 12th h from day 0 to day 6 in primary screen.

Day	PE											
	**P3 A1**	**P3 A2**	**P3 A3**	**P3 A4**	**P3 A5**	**P3 A6**	**P3 A7**	**P3 A8**	**P3 A9**	**P3 A10**	**P3 A11**	**P3 A12**
0	0.717	0.698	0.674	0.707	0.727	0.620	0.660	0.691	0.719	0.751	0.740	0.693
2	0.689	0.662	0.586	0.596	0.591	0.480	0.534	0.630	0.641	0.700	0.674	0.693
4	0.738	0.695	0.606	0.638	0.642	0.510	0.531	0.665	0.642	0.719	0.710	0.753
6	0.506	0.594	0.495	0.563	0.580	0.487	0.406	0.313	0.549	0.614	0.644	0.581
	**P3 B1**	**P3 B2**	**P3 B3**	**P3 B4**	**P3 B5**	**P3 B6**	**P3 B7**	**P3 B8**	**P3 B9**	**P3 B10**	**P3 B11**	**P3 B12**
0	0.725	0.754	0.747	0.709	0.748	0.749	0.666	0.515	0.755	0.757	0.753	0.745
2	0.658	0.668	0.676	0.615	0.503	0.666	0.654	0.679	0.697	0.661	0.661	0.626
4	0.742	0.708	0.715	0.678	0.701	0.676	0.680	0.700	0.670	0.680	0.676	0.736
6	0.512	0.651	0.644	0.572	0.360	0.592	0.502	0.543	0.624	0.659	0.631	0.621
	**P3 C1**	**P3 C2**	**P3 C3**	**P3 C4**	**P3 C5**	**P3 C6**	**P3 C7**	**P3 C8**	**P3 C9**	**P3 C10**	**P3 C11**	**P3 C12**
0	0.715	0.707	0.657	0.656	0.694	0.715	0.701	0.718	0.715	0.743	0.733	0.706
2	0.658	0.603	0.595	0.605	0.594	0.607	0.587	0.652	0.653	0.628	0.663	0.653
4	0.691	0.584	0.557	0.577	0.540	0.550	0.526	0.590	0.640	0.631	0.659	0.675
6	0.654	0.578	0.594	0.565	0.506	0.519	0.370	0.564	-0.002	-0.002	-0.003	-0.004
	**DMSO**											
0	0.728											
2	0.655											
4	0.641											
6	0.533											

Note: Green highlighted column(s) indicate absorbance values (A_{600}) which are higher than that of the DMSO control culture which is highlighted in blue. The PEs that have higher A_{600} throughout the ageing period (day 0, 2, 4, 6) are highlighted in orange.

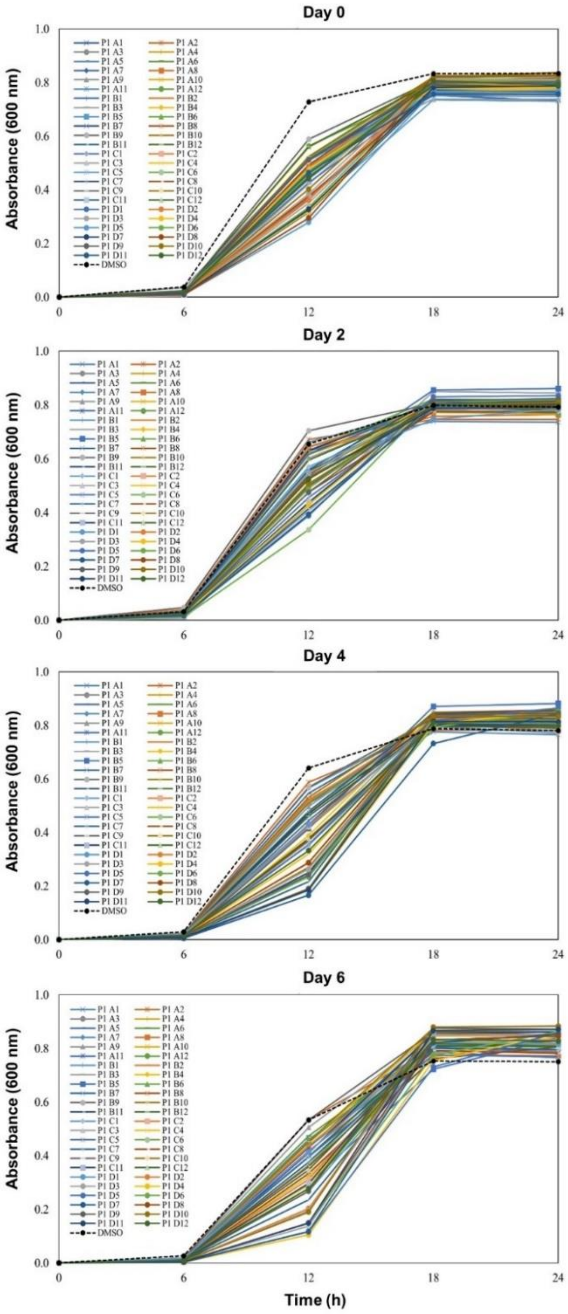

Figure S1. CLS outgrowth curves of yeast treated with PEs (P1 A1 until P1 D12) in primary screen. Yeast cells of strain 1783 treated with various PEs at final concentration of 100 μg/mL or DMSO control were aged in a 96-well microplate containing SC media for 8 days. A small aliquot of aged culture was transferred to a new 96- microplate containing fresh YPD medium for 24 h of outgrowth every two days of ageing. The absorbances (A_{600}) were recorded using Bio Microplate Reader HiTS every 6 h. Growth curves of absorbance against time of outgrowth were plotted. Three biological replicates of each culture were prepared.

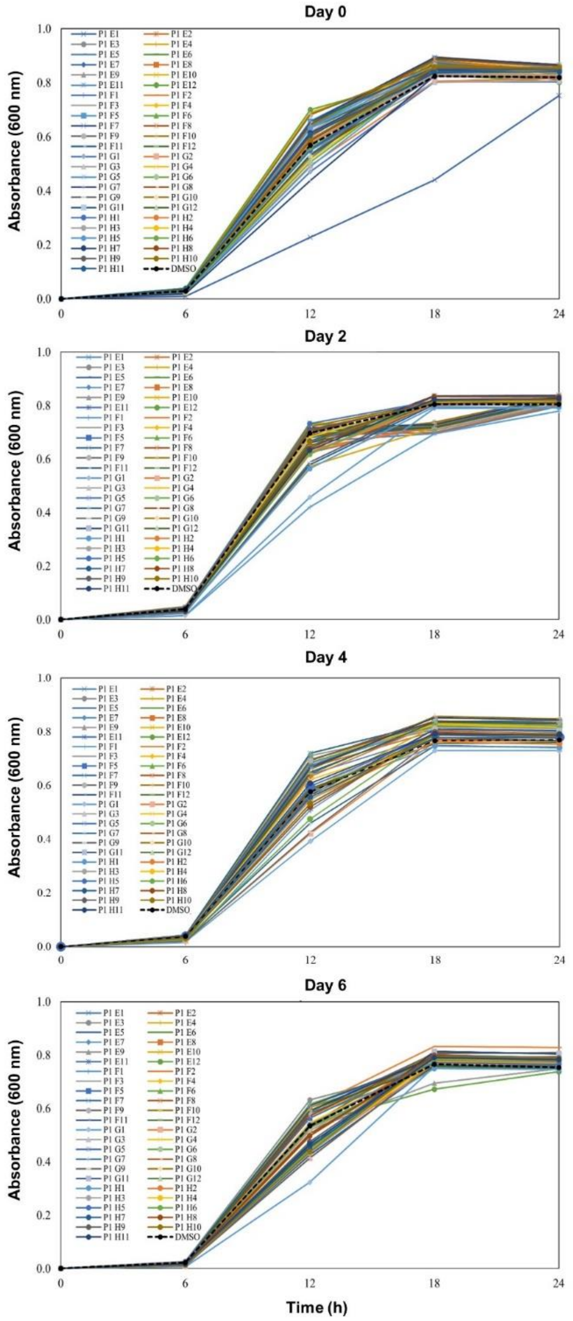

Figure S2. CLS outgrowth curves of yeast treated with PEs (P1 E1 till P1 H11) in primary screen. The CLS outgrowth method was repeated as described in Figure S1 for primary screen.

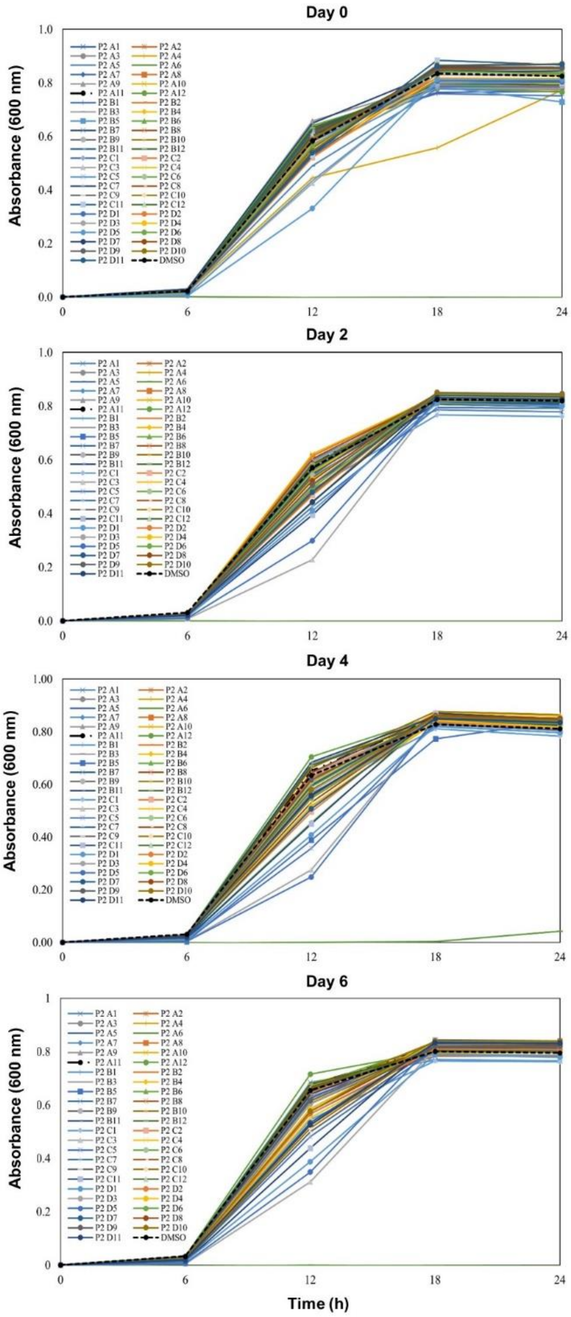

Figure S3. CLS outgrowth curves of yeast treated with PEs (P2 A1 until P2 D11) in primary screen. The CLS outgrowth method was repeated as described in Figure S1 for primary screen.

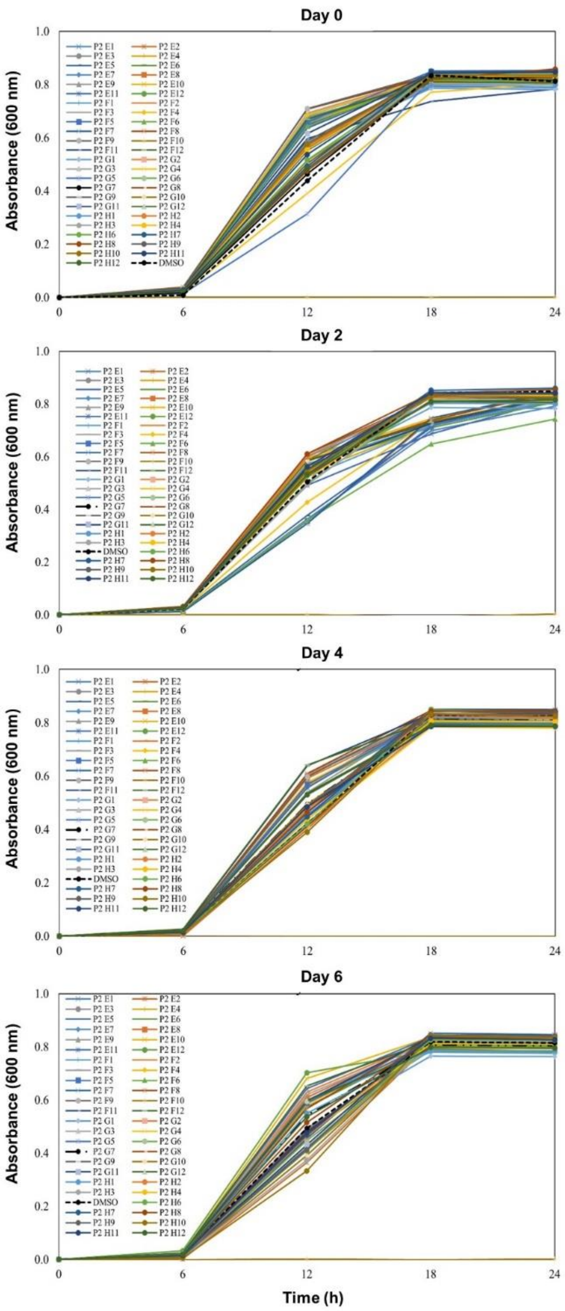

Figure S4. CLS outgrowth curves of yeast treated with PEs (P2 E1 until P2 H12) in primary screen. The CLS outgrowth method was repeated as described in Figure S1 for primary screen.

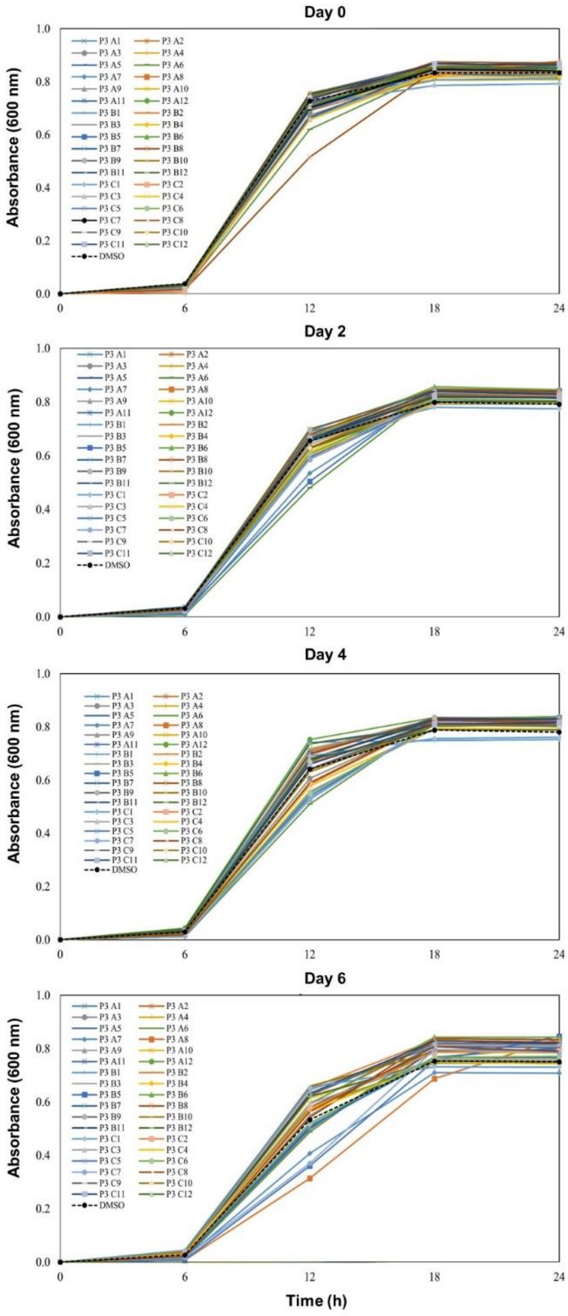

Figure S5. CLS outgrowth curves of yeast treated with PEs (P3 A1 until P3 C12) in primary screen. The CLS outgrowth method was repeated as described in Figure S1 for primary screen.

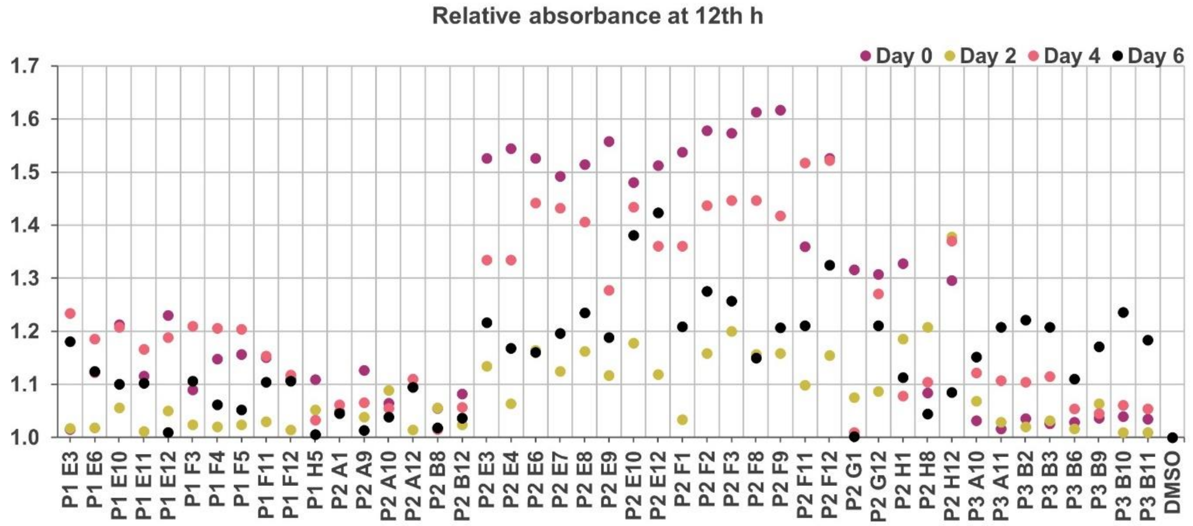

Figure S6. Relative absorbance values (A_{600}) of 45 PEs-treated cultures which are higher than that of the DMSO control culture at 12th h from day 0 to day 6 in primary screen. The CLS outgrowth method performed was described as in Figure S1 for primary screen. The relative absorbance values were calculated by dividing the PE-treated cultures' average outgrowth absorbance readings at 12th h to that of the DMSO control culture.

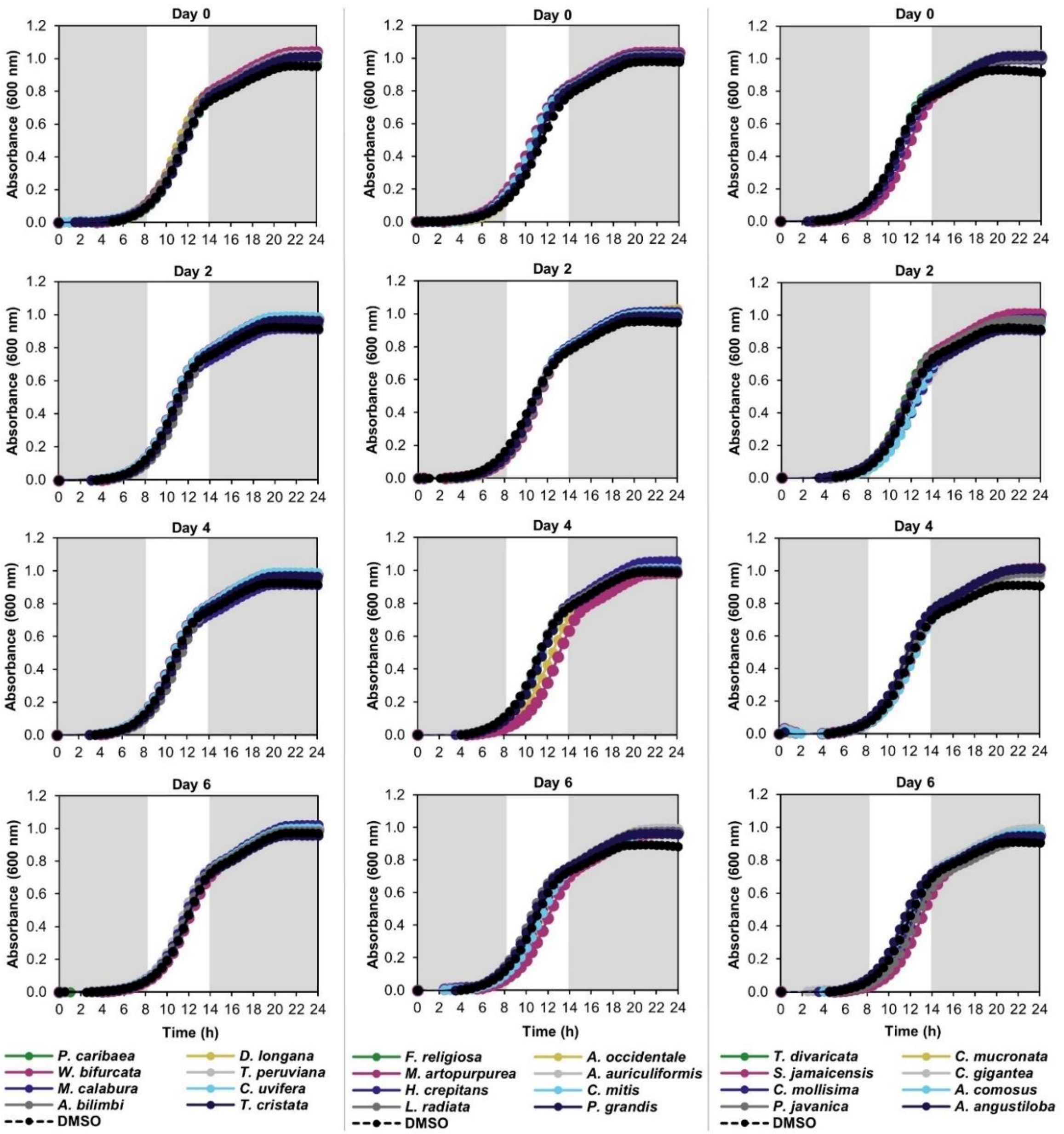

Figure S7. CLS outgrowth curves of yeast treated with PEs in secondary screen. Yeast cells of strain 1783 treated with various PEs selected from primary screen at final concentration of 100 μg/mL or DMSO control were aged in a 96-well microplate containing SC media for 8 days. A small aliquot of aged culture was transferred to a new 96- microplate containing fresh YPD medium for 24 h of outgrowth on every two days of ageing. The absorbances (A_{600}) were recorded using Bio Microplate Reader HiTS every 30 min up to 24 h. Growth curves of absorbance against time of outgrowth were plotted. Three biological replicates of each culture were prepared.

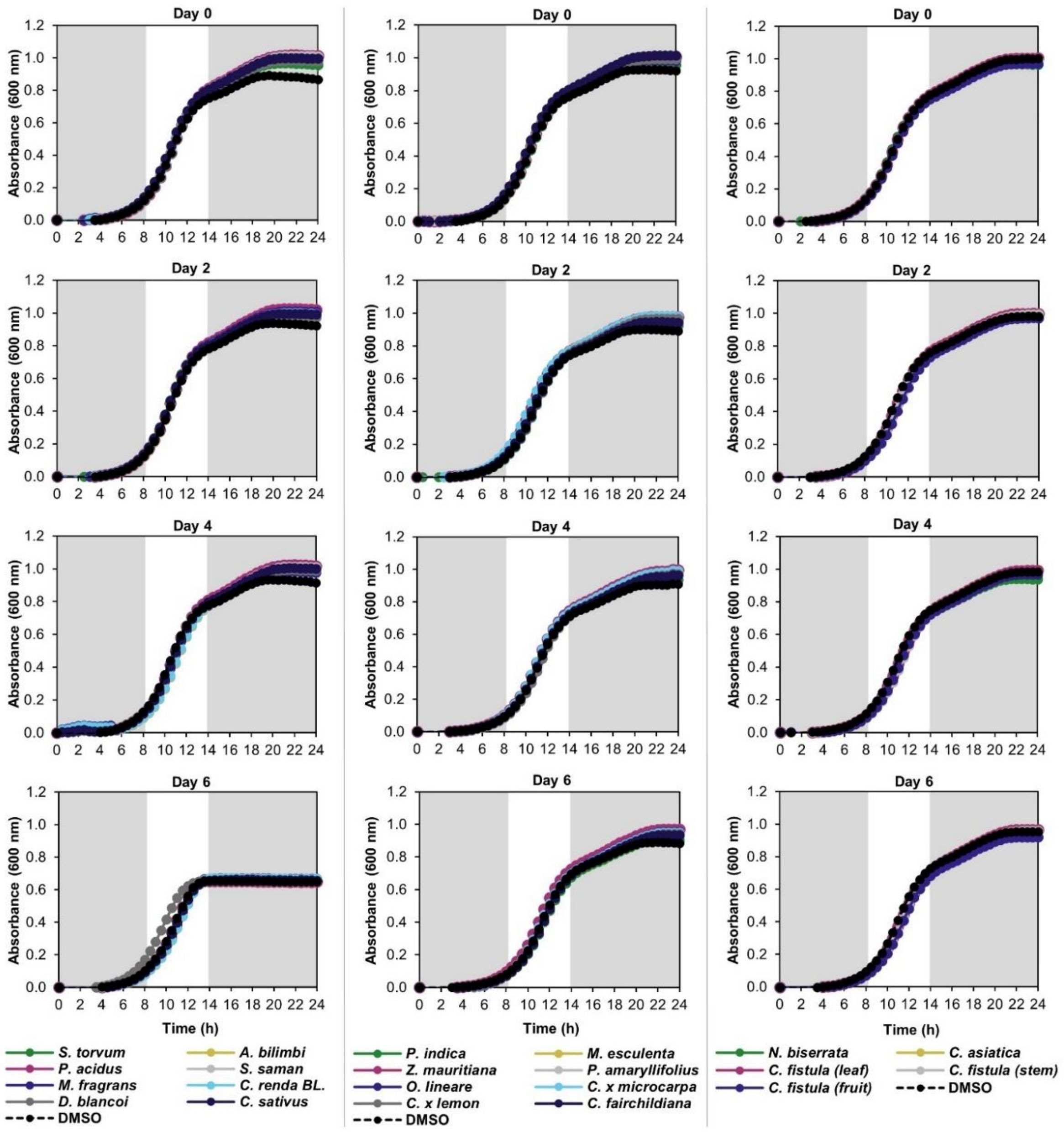

Figure S8. CLS outgrowth curves of yeast treated with PEs in secondary screen. The CLS outgrowth method was repeated as described in Figure S7 for secondary screen.

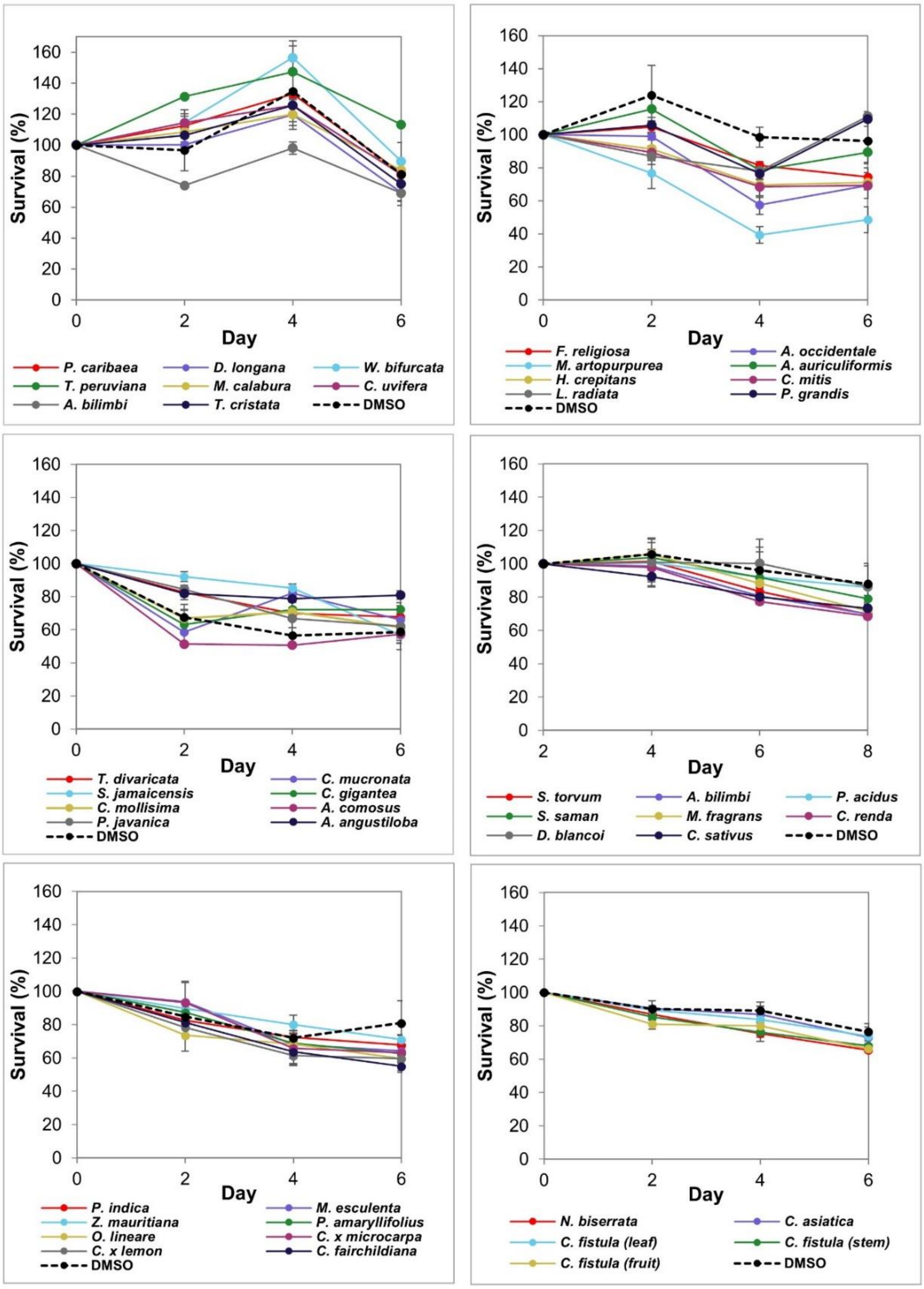

Figure S9. Survival of yeast treated with 45 PEs in secondary screen. The CLS outgrowth method was described as in Figure S7 for secondary screen. Absorbance readings (A_{600}) from Figure S7 and Figure S8 were analysed and converted to a survival curves. Error bars represent the SEM of three biological replicates.

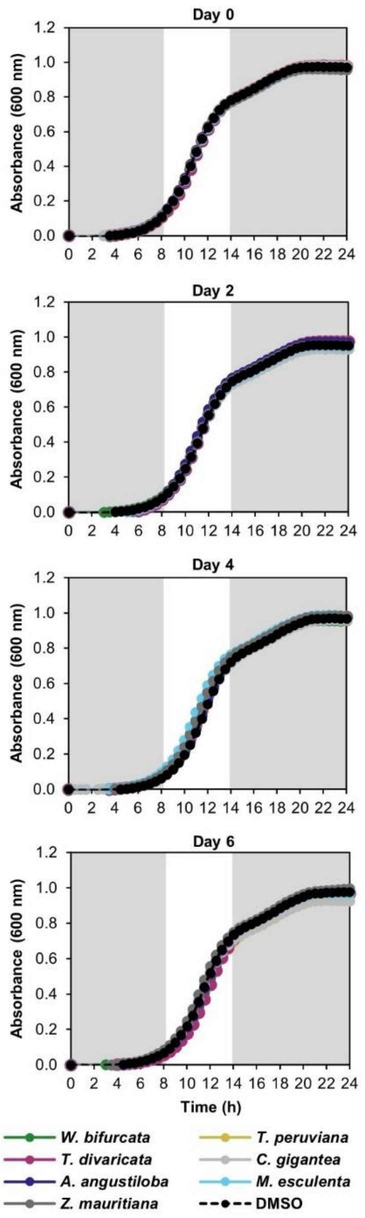

Figure S10. CLS outgrowth curves of yeast treated with seven PEs in confirmatory screen. Yeast cells of strain 1783 treated with various PEs selected from secondary screen at final concentration of 100 μg/mL or DMSO control were aged in a 96-well microplate containing SC media for 8 days. A small aliquot of aged culture was transferred to a new 96- microplate containing fresh YPD medium for 24 h of outgrowth on every two days of ageing. The absorbances (A_{600}) were recorded using Bio Microplate Reader HiTS every 30 min up to 24 h. Growth curves of absorbance against time of outgrowth were plotted. Three biological replicates of each culture were prepared.

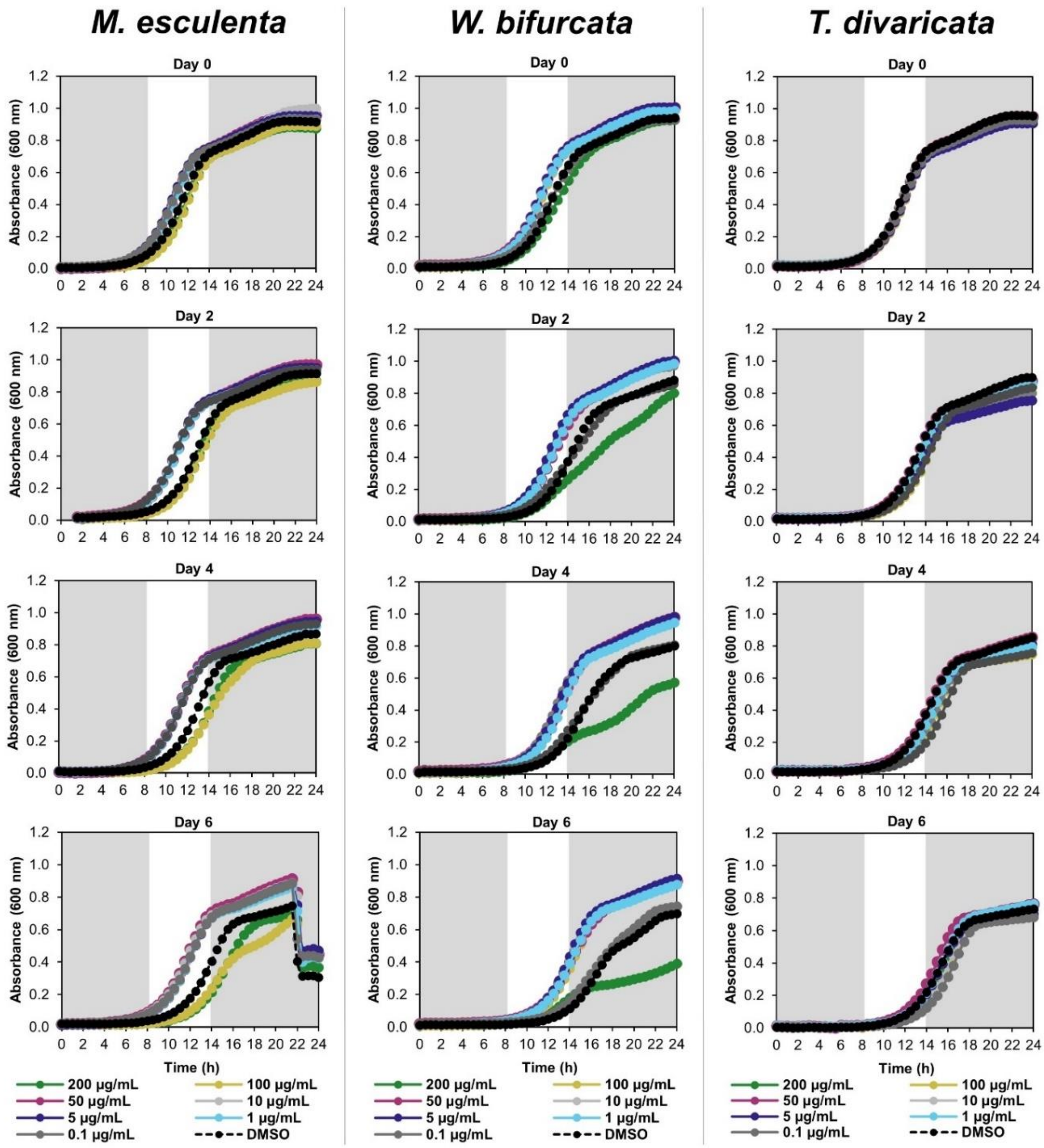

Figure S11. CLS outgrowth curves of yeast treated with three PEs in dose-dependent assay. Yeast cells of strain 1783 treated with *M. esculenta*, *W. bifurcata* or *T. divaricata* leaf extract in a range of concentrations (0.1 µg/mL – 200 µg/mL), or DMSO control were aged in bijou bottles containing SC media for 8 days. A small aliquot of each aged culture was transferred to a 96-well microplate containing YPD medium for 24 h of outgrowth on every two days of ageing. The absorbances (A_{600}) were recorded using Bio Microplate Reader HiTS every 30 min up to 24 h. Growth curves of absorbance against time of outgrowth were plotted. Three biological replicates of each culture were prepared.

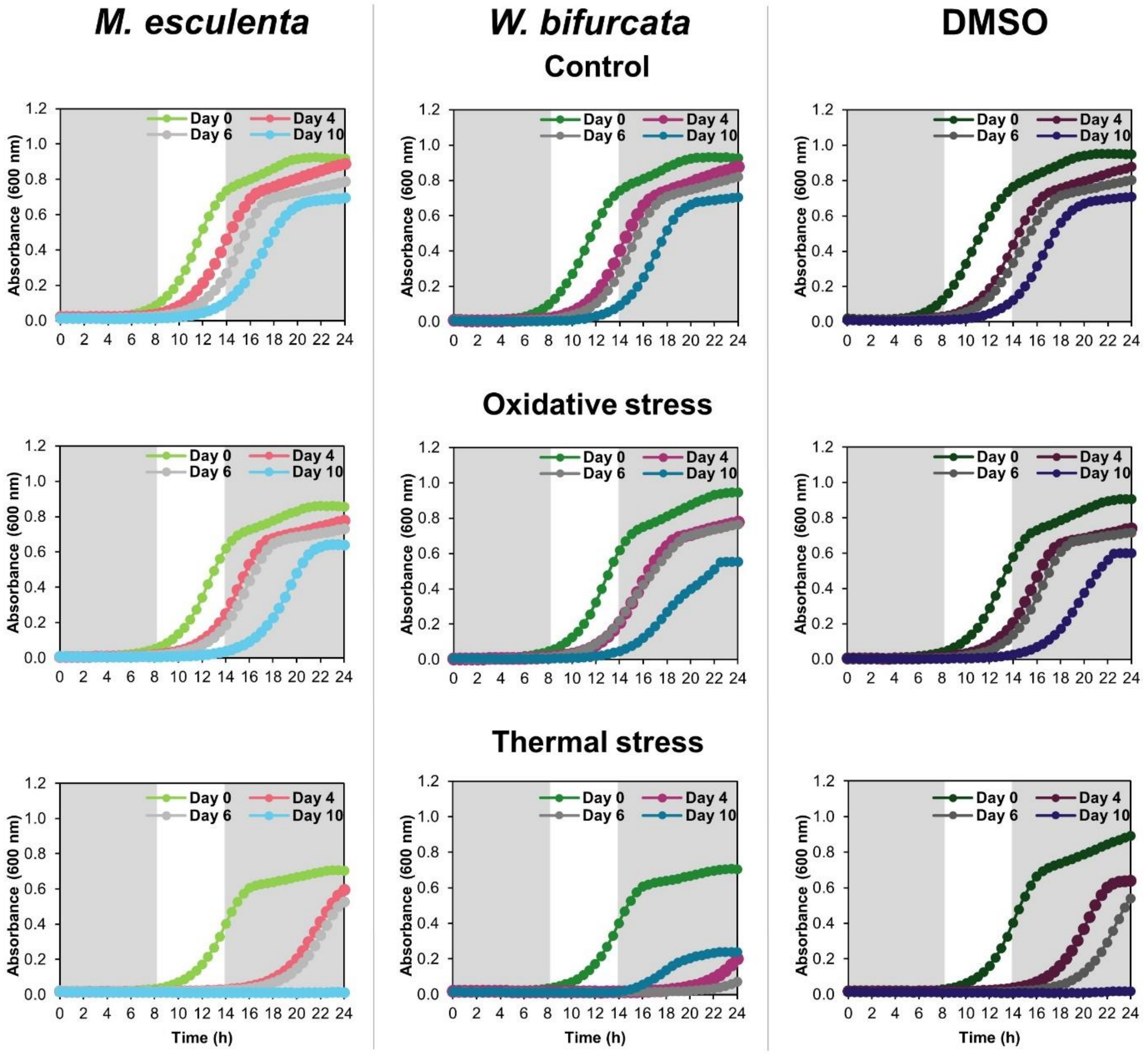

Figure S12. CLS outgrowth curves of yeast treated with two PEs in stress assays. Yeast cells of strain 1783 treated with *M. esculenta* or *W. bifurcata* leaf extract at optimal final concentration of 50 µg/mL and 10 µg/mL respectively or DMSO control were aged in bijou bottles for 12 days. At each age point, a small volume of aged yeast cells was exposed to oxidative stress by being treated with 3 mM H_2O_2 for 1 h at 30°C or thermal stress at 55°C for 20 min. An aliquot of each nonstress- or stress-treated culture was transferred to a 96-well microplate containing YPD medium for 24 h of outgrowth on day 0, 4, 6 and 10. The absorbances (A_{600}) were recorded using Bio Microplate Reader HiTS every 30 min up to 24 h. Growth curves of absorbance against time of outgrowth were plotted. Three biological replicates of each culture were prepared.
